# Diversity and Ethnobotany of the Family Zingiberaceae in Lop Buri Province, Thailand, with Notes on a Putative Natural Hybrid †

**DOI:** 10.3390/life16061023

**Published:** 2026-06-18

**Authors:** Thawatphong Boonma, Surapon Saensouk, Piyaporn Saensouk, Tepkanya Promkatkeaw

**Affiliations:** 1 Diversity of Family Zingiberaceae and Vascular Plant for Its Applications, Walai Rukhavej Botanical Research Institute, Mahasarakham University, Maha Sarakham 44150, Thailand; boonma.thawat@gmail.com; 2 Diversity of Family Zingiberaceae and Vascular Plant for Its Applications, Department of Biology, Faculty of Science, Mahasarakham University, Maha Sarakham 44150, Thailand; pcornukaempferia@yahoo.com; 3 Department of Science Education, Faculty of Science, Srinakharinwirot University, Bangkok 10110, Thailand; tepkanya@g.swu.ac.th

**Keywords:** *Curcuma*, endemic, ethnobotany, natural hybrid, traditional medicine, vascular plant diversity

## Abstract

Zingiberaceae is an ecologically, economically, and culturally important plant family in tropical Asia, yet its diversity and ethnobotanical significance remain insufficiently documented in several parts of Thailand. This study investigated the diversity, traditional uses, preliminary regional conservation status, and selected taxonomic aspects of Zingiberaceae in Lop Buri Province, central Thailand. Field surveys, herbarium studies, morphological comparisons, informal ethnobotanical interviews, quantitative use analyses, and preliminary conservation assessments were conducted from 2024 to 2025. A total of 110 taxa, comprising 109 species and one putative natural hybrid, were recorded in 13 genera and three tribes. These included wild, cultivated, introduced, and locally utilized taxa documented from natural habitats, home gardens, agricultural areas, and other human-associated habitats. Among them, 43 taxa were recorded from natural habitats as wild or naturally occurring populations, whereas 95 taxa were newly documented for Lop Buri Province. Natural habitats, particularly limestone areas, mixed deciduous forests, and dry evergreen forests, supported important native and endemic taxa. Ethnobotanical data from 110 informants documented 5113 use reports for 106 taxa, covering food, spice, medicinal, ornamental, ritual and belief-based, cosmetic, and commercial uses. *Curcuma* and *Alpinia* turned out to be the most frequently utilized genera. A putative natural hybrid, *Curcuma × lopburiensis*, is also described. These findings highlight Lop Buri Province as a hot spot for Zingiberaceae diversity and an important area for traditional plant knowledge and conservation in central Thailand.

## 1. Introduction

Zingiberaceae is one of the most diverse and economically important families of tropical monocotyledons, distributed predominantly throughout tropical and subtropical regions, particularly in Southeast Asia [1]. The family comprises approximately 1600 species in 59 accepted genera [2]. Species of Zingiberaceae possess considerable ecological, medicinal, culinary, ornamental, and cultural importance, and many taxa are widely utilized in traditional medicine, food preparation, spices, ritual practices, and horticulture [3,4,5,6,7]. In tropical ecosystems, members of the family also contribute to ecological processes such as pollinator interactions and forest regeneration [8,9].

Thailand represents one of the major centers of diversity of Zingiberaceae in mainland Southeast Asia, with over 390 species occurring across a wide range of vegetation types and ecological conditions [10]. The country is traditionally divided into seven floristic regions that differ in climate, geology, topography, and vegetation composition, all of which strongly influence species distribution patterns [11]. Recent botanical exploration has resulted in the discovery of numerous new species, new distributional records, and taxonomic updates within Thai Zingiberaceae [4,12,13,14], indicating that the diversity of the family remains incompletely documented in several parts of the country. Nevertheless, floristic and ethnobotanical information remains insufficient for many provinces, particularly in regions characterized by heterogeneous habitats and isolated limestone systems [15].

Lop Buri Province is situated within the Central Floristic Region of Thailand and contains a mosaic of habitats, including dry evergreen forests, mixed deciduous forests, dry dipterocarp forests, agricultural landscapes, and limestone hills [11,15,16]. Such environmental heterogeneity potentially supports considerable diversity of Zingiberaceae species. Habitat heterogeneity may facilitate species turnover and microhabitat specialization, while limestone ecosystems frequently harbor localized taxa. However, despite the publication of the Flora of Thailand [10] account for Zingiberaceae, only 14 wild species have previously been documented from Lop Buri Province based on available distribution records, suggesting that the naturally occurring diversity of the family in the province has likely been underestimated. Continued floristic surveys are therefore necessary to improve our understanding of species diversity, distribution patterns, and habitat associations of Zingiberaceae in central Thailand. Lop Buri Province was selected as a province-level case study rather than as a representative of Thailand as a whole. The province provides an important regional example within the Central Floristic Region because it contains heterogeneous habitats, especially limestone-associated landscapes, that may support localized, endemic, and previously underdocumented Zingiberaceae taxa. Therefore, the present study was designed to address a provincial knowledge gap and to contribute regional baseline data for future floristic, ethnobotanical, and conservation studies.

In addition to their floristic significance, species of Zingiberaceae are deeply associated with local ethnobotanical practices in Thailand. Many taxa are used as medicinal plants, food ingredients, spices, ornamentals, and cultural or ritual plants [3,4,7,12,15]. Traditional knowledge associated with these plants has accumulated through generations and represents an important component of local cultural heritage [15]. However, such knowledge is increasingly threatened by urbanization, socio-economic change, habitat degradation, deforestation, and the gradual loss of traditional lifestyles [3,4]. Documentation of ethnobotanical knowledge is therefore important not only for cultural preservation but also for supporting biodiversity conservation and the sustainable utilization of plant resources.

Habitat destruction and environmental disturbance are among the principal threats affecting many species of Zingiberaceae in Thailand, particularly taxa associated with localized habitats such as limestone ecosystems [15]. During field surveys conducted in Lop Buri Province, several noteworthy taxa were documented, including a small population of *Curcuma* showing morphological characters intermediate between *Curcuma saraburiensis* Boonma & Saensouk and *C. parviflora* Wall. [10]. The putative hybrid population was found growing near populations of both closely related species within the same area, and all three taxa belong to the same subgenus, *Hitcheniopsis* [17], suggesting the possibility of natural hybridization [18].

Therefore, the aims of the present study are: (1) to document the diversity, ethnobotanical uses, and preliminary regional conservation status of Zingiberaceae in Lop Buri Province, Thailand; and (2) to provide taxonomic notes and a description of a putative natural hybrid in *Curcuma*. Given the habitat heterogeneity of Lop Buri Province and the limited number of previously documented records, we expected that the diversity of Zingiberaceae in the province has been underestimated and that natural habitats, particularly limestone-associated areas, may support important native and endemic taxa. This study forms part of a broader investigation of Zingiberaceae diversity across multiple floristic regions of Thailand and contributes to the understanding of floristic diversity, traditional plant knowledge, and taxonomic relationships within the family.

## 2. Materials and Methods

### 2.1. Study Area

**Figure 1:**
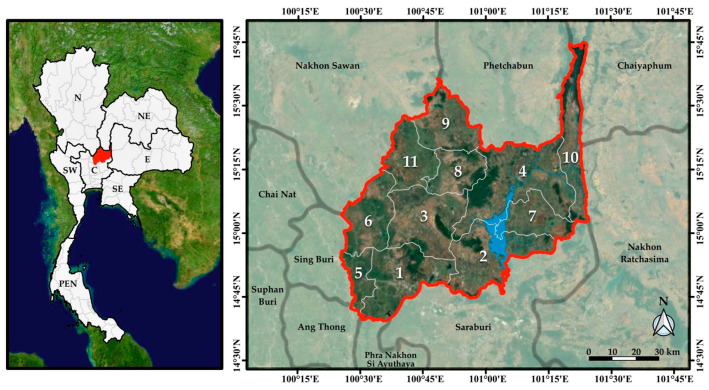
Floristic regions of Thailand and location of the study area. Left: the seven floristic regions of Thailand, with Lop Buri Province located in the Central Floristic Region. The red area indicates Lop Buri Province. Abbreviations: N = Northern, NE = Northeastern, E = Eastern, C = Central, SE = Southeastern, SW = Southwestern, and PEN = Peninsular. Right: study area in Lop Buri Province. The red line represents the provincial boundary, and the numbered areas correspond to the 11 districts: (1) Mueang Lop Buri, (2) Phatthana Nikhom, (3) Khok Samrong, (4) Chai Badan, (5) Tha Wung, (6) Ban Mi, (7) Tha Luang, (8) Sa Bot, (9) Khok Charoen, (10) Lam Sonthi, and (11) Nong Muang. The blue is water in Pasak Jolasid Dam.

Lop Buri Province is located in central Thailand within the Central Floristic Region and shares boundaries with eight neighboring provinces (Figure 1). The province is characterized by diverse topography consisting of lowland floodplains, undulating plains, upland areas, and limestone formations. Extensive alluvial plains associated with the Lop Buri and Pa Sak river systems occupy a substantial portion of the province, whereas upland landscapes and foothills connected to the Phetchabun Mountain Range dominate the eastern region. Forested areas account for approximately 962 km^2^ or about 14.8% of the provincial area. The climate is classified as tropical savanna, with a pronounced dry period from November to April and a rainy season from May to October, corresponding largely to the southwest monsoon period. The highest temperatures are typically recorded during April, while cooler conditions generally occur between December and January. Most precipitation is concentrated during the monsoon season, especially in September and October. Natural vegetation in the province includes dry evergreen forests, mixed deciduous forests, dry dipterocarp forests, agricultural landscapes, and limestone-associated habitats. These heterogeneous environmental conditions potentially support substantial diversity of Zingiberaceae species [19]. Botanical surveys were conducted across multiple localities throughout the province between 2024 and 2025. During each field investigation, habitat characteristics, elevation, associated vegetation, and general locality information were recorded.

### 2.2. Field Surveys and Specimen Collection

Field investigations were conducted from 2024 to 2025, in accordance with local regulations. When collections were made on private or community land, permission was obtained from landowners or local community representatives prior to fieldwork. No collections were made in strictly protected areas without permission. Surveys were undertaken at least one to two times per month throughout the study period. Plant specimens were collected and processed following standard herbarium procedures [20]. Voucher specimens were deposited at the Vascular Plant Herbarium, Mahasarakham University (VMSU).

The occurrence records reported for Lop Buri Province in this study were based primarily on field-collected specimens and field observations made by the authors within the province. Specimens from other herbaria were used only as comparative materials for taxonomic identification and nomenclatural verification and were not treated as primary occurrence records for Lop Buri Province unless supported by the present field survey.

Generally, a single voucher specimen was prepared for each species encountered during the study. However, specimens displaying unusual or distinct morphological variation were collected separately and assigned independent collection numbers for additional taxonomic examination. During the surveys, one population of *Curcuma* exhibiting morphological characters intermediate between *Curcuma saraburiensis* and *C. parviflora* was discovered growing sympatrically with both taxa. Additional collections and detailed observations were therefore undertaken to assess its taxonomic status and compare it with previously described taxa [10].

### 2.3. Taxonomic Study

Morphological observations were undertaken using freshly collected materials together with preserved herbarium specimens. Measurements of vegetative and reproductive structures were obtained using a standard ruler and a Mitutoyo vernier caliper (Mitutoyo Corporation, Kawasaki, Japan). Detailed examinations of floral morphology, surface texture, and inflorescence structures were carried out under a stereoscopic microscope (Stemi 2000-C; Zeiss, Oberkochen, Germany).

Taxonomic identification was based on comparisons with living plants, herbarium specimens, protologues, and relevant taxonomic literature, including treatments published in the Flora of Thailand Volume 16 Part 2 [10]. Morphological terminology followed Beentje [21]. Scientific names and nomenclatural information were verified using Plants of the World Online [2] and International Plant Names Index [22], whereas herbarium acronyms followed the standards of Index Herbariorum [23]. The institutional abbreviations used in this study are as follows: Harvard University, Cambridge, Massachusetts, USA (A), Aarhus University, Denmark (AAU), Botanic Garden and Botanical Museum Berlin-Dahlem, Germany (B), Bangkok Herbarium, Department of Agriculture, Thailand (BK), Department of National Parks, Wildlife and Plant Conservation, Thailand (BKF), The Natural History Museum, London, England, UK (BM), University of Copenhagen, Copenhagen, Denmark (C), Botanical Survey of India, Howrah, West Bengal, India (CAL), Medicinal Plants Research Institute, Thailand (DMSC), Royal Botanic Garden Edinburgh, Edinburgh, UK (E), Xishuangbanna Tropical Botanical Garden, Academia Sinica, Yunnan, China (HITBC), Vietnam Academy of Science and Technology (VAST), Hanoi, Vietnam (HN), National Institute of Medicinal Materials, Vietnam (HNPM), VNU University of Science, Hanoi, Vietnam (HNU), Guangxi Institute of Botany, China (IBK), Chinese Academy of Medical Sciences, China (IMDY), Royal Botanic Gardens, UK (K), Khon Kaen University Herbarium, Thailand (KKU), Naturalis Biodiversity Center, Leiden, Netherlands (L), The New York Botanical Garden, New York, USA (NY), Muséum National d’Histoire Naturelle, Paris, France (P), Queen Sirikit Botanic Garden, The Botanical Garden Organization, Thailand (QBG), Southern Institute of Ecology, Ho Chi Minh City, Vietnam (SGN), Singapore Botanic Gardens, National Parks Board, Singapore (SING), National Museum of Nature and Science, Tokyo, Japan (TNS), Vascular Plant Herbarium, Mahasarakham University, Thailand (VMSU), Institute of Life Science, Ho Chi Minh City, Vietnam (VNM), and Vietnam National Museum of Nature, Hanoi, Vietnam (VNMN).

Diagnostic characters used for identification included habit, rhizome morphology, root characters, leaf sheath, ligule, petiole, lamina shape and indumentum, inflorescence position, bract and bracteole morphology, calyx, floral tube and lobes, labellum, lateral staminodes, anther, ovary, filament, stigma, color pattern, phenology, habitat, and distribution. Identification was finalized only after comparison with relevant protologues, taxonomic keys, herbarium specimens, living collections, and published taxonomic treatments. Taxonomic comparisons were based on both physical herbarium specimens and digitized collections available through online repositories. Herbarium materials accessible for direct examination were studied on-site, whereas additional specimens were investigated using high-resolution digital images and online databases, including the Global Biodiversity Information Facility (GBIF; GBIF.org. 2025. GBIF Occurrence Download. Available online: https://doi.org/10.15468/dl.4hvbcm (accessed on 27 July 2025)). Specimens examined directly during this study originated from the following herbaria: A, AAU, B, BK, BKF, BM, C, CAL, DMSC, E, HN, HNPM, HNU, IBK, IMDY, K, KKU, L, P, QBG, SGN, SING, VMSU, VNM, and VNMN. Additional materials examined exclusively through online resources were obtained from HITBC, NY, and TNS.

The putative hybrid was evaluated based on comparative morphological investigation, sympatric occurrence of the presumed parental taxa observed in the field, and the presence of intermediate morphological characters between *Curcuma saraburiensis* and *C. parviflora*.

### 2.4. Morphometric Analysis of the Putative Hybrid

Morphometric comparisons among the putative hybrid detected in this study and its presumed parental taxa, *Curcuma saraburiensis* and *C. parviflora*, were conducted using mature flowering individuals observed during the same flowering season in order to minimize variation associated with developmental stage and seasonal effects. Morphometric analysis included 20 individuals of *C. saraburiensis*, 20 individuals of *C. parviflora*, and 5 individuals of the putative hybrid, reflecting the limited number of individuals encountered during field surveys. Measurements were obtained from fully developed vegetative shoots and freshly opened flowers.

Quantitative characters selected for analysis included leafy shoot height, rhizome length and width, number of leaves per leafy shoot, leaf blade length and width, petiole length, peduncle length, thyrse length, fertile bract length and width, calyx length, floral tube length, labellum length and width, labellum incision depth, lateral staminode length and width, and anther length.

Principal Component Analysis (PCA) was performed using a correlation matrix in PAST version 4.15 [24]. Qualitative and semi-quantitative characters, including color patterns, indumentum, and floral ornamentation, were excluded from the PCA and instead evaluated separately through comparative morphological assessment.

### 2.5. Ethnobotanical Data Collection

Ethnobotanical information was obtained through informal interviews with local informants from 11 districts of Lop Buri Province. Informants were purposively selected based on their familiarity with local plant resources and their practical knowledge of traditional plant uses. Most were older members of local communities who had accumulated knowledge through long-term use, observation, and transmission of local practices. A total of 110 informants participated in the study. Before each interview, the objectives of the research and the scope of the questions were explained to all participants. Only information associated with traditional plant uses and local knowledge was documented, and no personal or sensitive information was recorded. Interviews were conducted only after verbal informed consent had been obtained from the participants.

Ethnobotanical records compiled during interviews included vernacular names, ethnolinguistic information, traditional uses, plant parts utilized, preparation methods, seasonal availability, and sources of plant materials, including wild collection, cultivation, and market purchase. Recorded uses were categorized into food, spice, medicinal, ornamental, ritual and belief, cosmetic, and commercial uses [3,15]. Medicinal applications were further categorized according to the 17 principal therapeutic groups proposed by the National Drug System Development Committee [25].

Ethnobotanical information was recorded for Zingiberaceae taxa encountered and identified during the present study, regardless of whether they had previously been documented as wild species from Lop Buri Province [10]. Some useful taxa were obtained from natural habitats, whereas others were cultivated in home gardens, maintained in agricultural areas, purchased from local markets, or introduced from nearby provinces and subsequently grown by local people. Therefore, the ethnobotanical component of this study reflects the current local use and management of Zingiberaceae in Lop Buri Province, rather than only the previously published wild distribution records in the Flora of Thailand [10].

### 2.6. Ethnobotanical Quantitative Analysis

#### 2.6.1. Species Use Value (SUV)

SUV was used to evaluate the relative ethnobotanical importance of each species following Phillips and Gentry [26] and Hoffman and Gallaher [27]. SUV was calculated using the formula:

(1) $$SUV=\frac{\sum U_{i}}{N}$$

where $U_{i}$ represents the total number of use reports recorded for a species and N represents the total number of informants.

#### 2.6.2. Genus Use Value (GUV)

GUV was calculated to assess the relative ethnobotanical importance of each genus based on the cumulative use values of species belonging to the same genus [26,27,28].

(2) $$GUV=\frac{\sum SUV}{N_{s}}$$

where ∑SUV is the sum of species use values within a genus and $N_{s}$ is the total number of species within that genus.

#### 2.6.3. Relative Frequency of Citation (RFC)

RFC was employed to evaluate the frequency with which each species was cited by informants [29].

(3) $$RFC=\frac{FC}{N}$$

where FC represents the number of informants mentioning a particular species and N represents the total number of informants.

#### 2.6.4. Plant Part Value (PPV)

PPV was used to evaluate the relative importance of plant parts utilized in ethnobotanical practices following Gomez-Beloz [30].

(4) $$PPV=\frac{{\sum RU}_{plant\;parts}}{\sum RU}\times 100$$

where RU_plant parts_ represents the number of use reports for a particular plant part and ∑RU represents the total number of use reports recorded for all plant parts.

#### 2.6.5. Informant Consensus Factor (Fic)

Fic was calculated to evaluate the degree of agreement among informants regarding the medicinal uses of plant species [31,32].

(5) $$Fic=\frac{n_{ur}-n_{t}}{n_{ur}-1}$$

where $n_{ur}$ represents the total number of use reports recorded for a particular therapeutic category and $n_{t}$ represents the number of taxa used for that category.

#### 2.6.6. Jaccard’s Similarity Index and Cluster Analysis

Similarity in species composition among habitat types was evaluated using Jaccard’s Similarity Index (JI) [33]. The index was calculated as:

(6) $$JI=\frac{a}{a+b+c}$$

where a represents the number of species shared between two habitat types, b represents the number of species unique to the first habitat type, and c represents the number of species unique to the second habitat type. Cluster analysis was subsequently performed using the Unweighted Pair Group Method with Arithmetic Mean (UPGMA) [34] based on the Jaccard similarity matrix. Dendrogram construction and clustering analyses were conducted using PAST software (version 4.15) [24].

### 2.7. Conservation Assessment

Conservation assessments were conducted based on field observations, habitat conditions, distribution range, estimated population size, and observed threats following the IUCN Red List Categories and Criteria [35,36]. For threatened or narrowly distributed taxa, especially those proposed as CR or EN, fieldwork was conducted with minimal disturbance. Only necessary voucher materials were collected for taxonomic verification, and destructive collection from small populations was avoided. Precise coordinates of threatened taxa are not disclosed in the manuscript in order to reduce the risk of illegal or excessive collection.

### 2.8. Data Visualization and Statistical Analysis

Descriptive statistical analyses were used to summarize species diversity, ethnobotanical uses, plant parts utilized, seasonal availability, and use categories of Zingiberaceae species recorded in Lop Buri Province. Frequencies and percentages of use reports were summarized in tables and figures. The proportions of wild-collected, cultivated, endemic, native, and introduced taxa were also evaluated based on field observations and literature records.

Ethnobotanical data obtained from interviews and field observations were categorized according to plant parts utilized, preparation methods, and use categories. Relationships among these variables were visualized using a Sankey diagram to illustrate the interconnections and flow of ethnobotanical knowledge associated with Zingiberaceae species in Lop Buri Province.

All graphical materials and figure plates were prepared using Pixelmator Pro version 3.6.5 (“Archipelago”) (Pixelmator Team, Vilnius, Lithuania) on a MacBook Pro (13-inch, M1, 2020; Apple Inc., Cupertino, CA, USA). Scientific illustrations were digitally prepared by Thawatphong Boonma using an Apple iPad Air (5th generation) running iPadOS 17.5.1.

## 3. Results

### 3.1. Diversity of Zingiberaceae in Lop Buri Province

A total of 110 taxa of Zingiberaceae were recorded from Lop Buri Province, comprising 109 species and one putative natural hybrid (nothosp. nov.), belonging to 13 genera (Table 1). Among these, 100 taxa were considered native to Thailand, whereas 10 species were recognized as introduced species. Forty-three taxa are endemic to Thailand. The genus *Curcuma* L. was the most species-rich genus recorded in the study area. The recorded taxa were distributed across various habitat types, including dry evergreen forests, limestone areas, mixed deciduous forests, agricultural areas, and home gardens. Limestone-associated habitats supported particularly high diversity, with 36 taxa recorded from limestone areas and adjacent karst habitats. In addition, the present study documented 95 taxa as newly recorded for Lop Buri Province, increasing the known diversity of Zingiberaceae in the region. Among the 110 taxa recorded in the present study, 43 taxa were documented from natural habitats as wild or naturally occurring populations. This represents a substantial increase in documented wild Zingiberaceae records for Lop Buri Province compared with the 14 wild species previously reported in the Flora of Thailand [10]. However, the total of 110 taxa includes not only wild taxa but also cultivated, introduced, and locally utilized taxa recorded from home gardens, agricultural areas, markets, and other human-associated habitats. Therefore, the increase reported here should be interpreted as an expansion of documented provincial knowledge rather than a recent increase in the actual number of taxa occurring naturally in the province.

**Figure 2:**
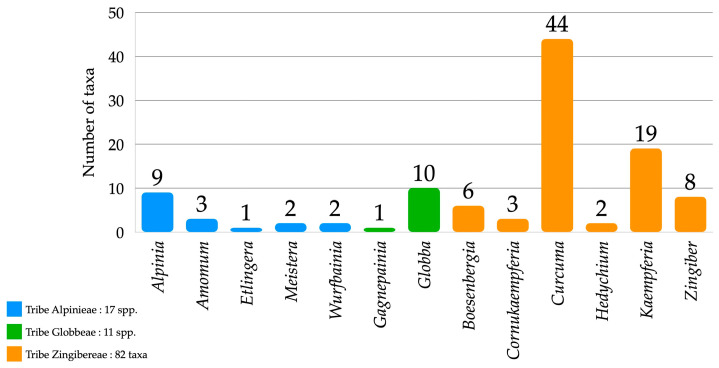
Diversity of Zingiberaceae taxa grouped by genera and tribes represented by different colors.

The diversity of Zingiberaceae in Lop Buri Province comprised 13 genera belonging to three tribes: Alpinieae, Globbeae, and Zingibereae (Figure 2). Tribe Zingibereae was the most species-rich group, accounting for 82 taxa, followed by Alpinieae with 17 species and Globbeae with 11 species. Among the recorded genera, *Curcuma* was the most diverse with 44 taxa, representing the largest proportion of the family in the study area. Other species-rich genera included *Kaempferia* (19 spp.), *Globba* (10 spp.), and *Zingiber* (8 spp.). Moderate diversity was observed in *Boesenbergia* (6 taxa), whereas *Alpinia* and *Cornukaempferia* each comprised 3 taxa. The remaining genera, namely *Amomum*, *Meistera*, *Wurfbainia*, and *Hedychium*, were represented by 2 taxa each, while *Etlingera* and *Gagnepainia* were the least diverse, with only 1 taxon recorded for each genus.

#### Distribution Patterns and Occurrence Types of Zingiberaceae

**Figure 3:**
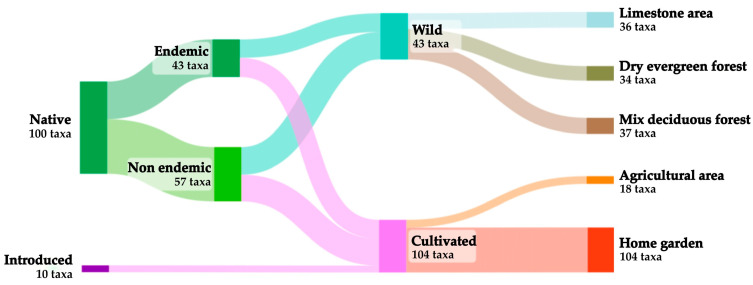
Sankey diagram of the distribution status, occurrence types, and habitat associations of Zingiberaceae taxa in Lop Buri Province, Thailand.

A total of 110 taxa of Zingiberaceae were recorded in Lop Buri Province, including 100 native taxa and 10 introduced taxa (Figure 3). Among the native taxa, 43 were endemic, and 57 were non-endemic. Wild taxa were mainly associated with mixed deciduous forest (37 taxa), limestone areas (36 taxa), and dry evergreen forest (34 taxa), whereas cultivated taxa were predominantly found in home gardens (104 taxa) and agricultural areas (18 taxa).

Forty-three taxa were recorded from natural habitats as wild or naturally occurring populations, whereas cultivated taxa were mainly associated with home gardens, agricultural areas, and other human-associated habitats. The total of 110 taxa includes wild, cultivated, introduced, and locally utilized taxa documented during the present survey.

**Figure 4:**
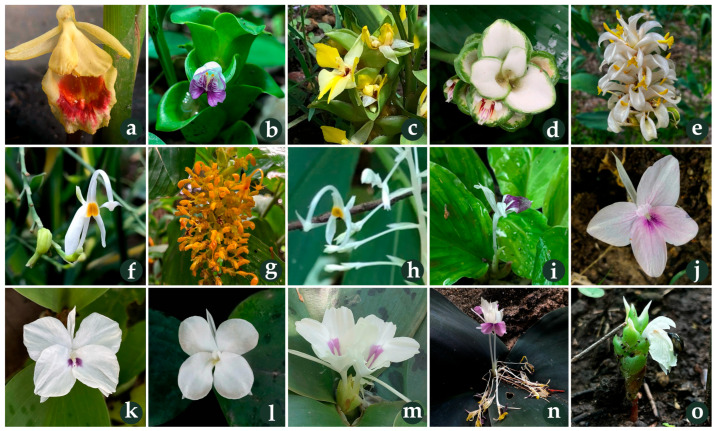
Wild and endemic Zingiberaceae species recorded from natural habitats in Lop Buri Province, Thailand: (**a**) *Boesenbergia collinsii*; (**b**) *Curcuma charanii*; (**c**) *C. putii*; (**d**) *C. saraburiensis*; (**e**) *C. supraneeana*; (**f**) *Globba chrysochila*; (**g**) *G. colpicola*; (**h**) *G. xantholeuca*; (**i**) *Kaempferia chaveerachiae*; (**j**) *K. lopburiensis*; (**k**) *K. maculifolia*; (**l**) *K. napavarniae*; (**m**) *K. pardi*; (**n**) *K. saraburiensis*; and (**o**) *Zingiber brachystachys*. All photographs by Thawatphong Boonma.

Among the native taxa, 15 species were both endemic to Thailand and recorded from natural habitats in Lop Buri Province (Figure 4). These taxa were mainly found in limestone areas, mixed deciduous forest, and dry evergreen forest. Most endemic wild taxa were associated with limestone habitats, whereas fewer taxa were recorded from disturbed or cultivated areas.

### 3.2. Ethnobotanical Uses of Zingiberaceae

**Figure 5:**
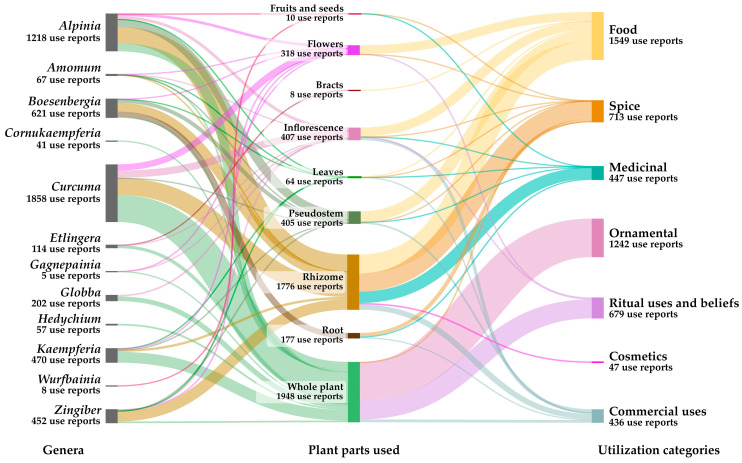
Sankey diagram of the relationships among genera of Zingiberaceae, plant parts used, and utilization categories recorded in Lop Buri Province, Thailand. The width of each flow corresponds to the number of ethnobotanical use reports documented from local informants.

Among the 110 recorded taxa of Zingiberaceae in Lop Buri Province, 106 taxa belonging to 12 genera were reported to possess ethnobotanical uses by local communities (Figure 5). These included *Alpinia* (9 spp.), *Amomum* (2 spp.), *Boesenbergia* (6 spp.), *Cornukaempferia* (3 spp.), *Curcuma* (44 taxa), *Etlingera* (1 sp.), *Gagnepainia* (1 sp.), *Globba* (10 spp.), *Hedychium* (2 spp.), *Kaempferia* (19 spp.), *Wurfbainia* (1 sp.), and *Zingiber* (8 spp.). A total of 5113 use reports were documented from interviews with 110 local informants. The recorded utilization categories included food, spice, medicinal, ornamental, ritual uses and beliefs, cosmetic, and commercial uses. Among these, food-related uses represented the highest number of reports (1549 use reports), followed by ornamental uses (1242 use reports), spice uses (713 use reports), ritual uses and beliefs (679 use reports), medicinal uses (447 use reports), commercial uses (436 use reports), and cosmetic uses (47 use reports).

The genera *Curcuma* (1858 use reports) and *Alpinia* (1218 use reports) were the most frequently utilized genera. Rhizomes (1776 use reports) and whole plants (1948 use reports) were the most commonly utilized plant parts, reflecting the important role of Zingiberaceae species in local food systems, traditional healthcare, ornamental cultivation, and cultural practices. Nevertheless, four species recorded during the present study, namely *Amomum repoeense*, *Meistera koenigii*, *M. tomrey*, and *Wurfbainia uliginosa*, were not associated with any reported ethnobotanical use by local informants.

#### 3.2.1. Used as Food

**Figure 6:**
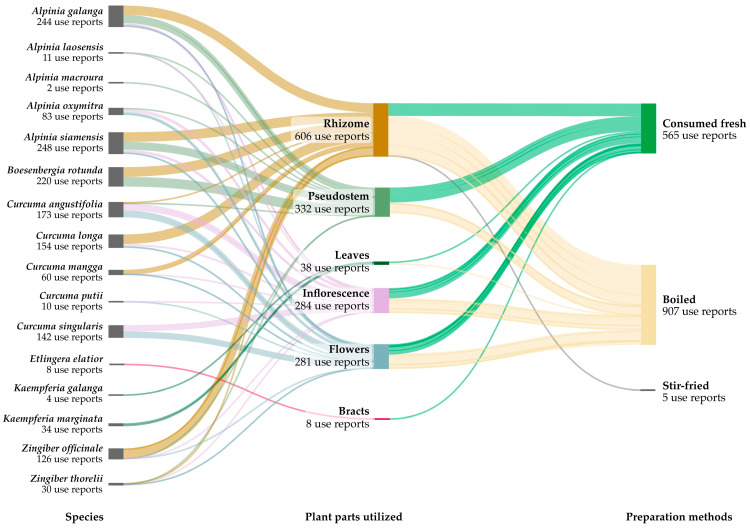
Sankey diagram of the relationships among Zingiberaceae species used as food, plant parts utilized, and preparation methods in Lop Buri Province, Thailand.

Food uses represented one of the most prominent utilization categories recorded during the present study (Figure 5). Numerous species of Zingiberaceae were consumed as vegetables, ingredients, or local food resources, particularly members of the genera *Alpinia*, *Boesenbergia*, *Curcuma*, and *Zingiber*. Rhizomes, inflorescences, flowers, pseudostems, leaves, and bracts were commonly utilized either fresh or cooked in local dishes. Species such as *Alpinia galanga*, *A. siamensis*, *Boesenbergia rotunda*, *Curcuma angustifolia*, *C. longa*, and *Zingiber officinale* were among the most frequently reported edible taxa (Figure 6). Rhizomes constituted the most frequently used plant part, accounting for 606 use reports, followed by pseudostems (332 use reports), inflorescences (284 use reports), and flowers (281 use reports). Most edible plant materials were consumed after boiling (907 use reports), whereas fresh consumption was also common (565 use reports), particularly for inflorescences, flowers, pseudostems, and young rhizomes. Stir-frying was comparatively rare, with only five use reports. Among the taxa documented, *Alpinia galanga*, *A. siamensis*, *Boesenbergia rotunda*, *Curcuma angustifolia*, and *Zingiber officinale* showed the highest diversity of edible plant parts and preparation methods, reflecting their importance in local culinary traditions.

#### 3.2.2. Used as Spice

**Figure 7:**
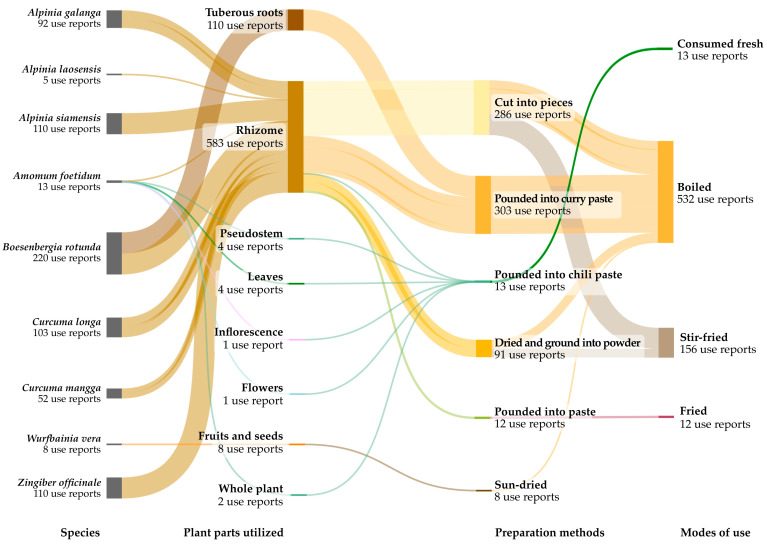
Sankey diagram of the relationships among Zingiberaceae species used as spice, plant parts utilized, and preparation methods in Lop Buri Province, Thailand.

Spice-related applications were recorded from eight Zingiberaceae species during the present study (Figure 7). *Boesenbergia rotunda* showed the highest number of spice-related use reports (220 use reports), followed by *Alpinia siamensis* and *Zingiber officinale* (110 use reports each), *Curcuma longa* (103 use reports), *Alpinia galanga* (92 use reports), *Curcuma mangga* (52 use reports), *Amomum foetidum* (13 use reports), *Wurfbainia vera* (8 use reports), and *Alpinia laosensis* (5 use reports). Rhizomes were the principal plant part utilized as spice, accounting for 583 use reports, followed by tuberous roots (110 use reports). Other plant parts were only occasionally recorded, including fruits and seeds (8 use reports), pseudostems (4 use reports), leaves (4 use reports), whole plants (2 use reports), flowers (1 use report), and inflorescences (1 use report). The recorded preparation methods included pounding into curry paste (303 use reports), cutting into pieces (286 use reports), drying and grinding into powder (91 use reports), pounding into chili paste (13 use reports), pounding into paste (12 use reports), and sun-drying (8 use reports). Most spice preparations were associated with boiling (532 use reports), followed by stir-frying (156 use reports), consumption in fresh form (13 use reports), and frying (12 use reports).

Several preparation methods were documented, with rhizomes commonly cut into pieces, pounded into curry paste, dried and ground into powder, or incorporated into chili paste preparations. Among these, pounding into curry paste represented the most frequently recorded preparation method (303 use reports), followed by cutting into pieces (286 use reports) and drying and grinding into powder (91 use reports). Most spice materials were subsequently used in boiled dishes (532 use reports), whereas stir-frying was also common (156 use reports). Fried preparations and fresh consumption were comparatively infrequent. Species such as *Alpinia galanga*, *Boesenbergia rotunda*, *Curcuma longa*, and *Zingiber officinale* showed the highest number of spice-related use reports, highlighting their major importance in local cuisine and traditional seasoning practices.

#### 3.2.3. Used for Herbal Medicine

**Figure 8:**
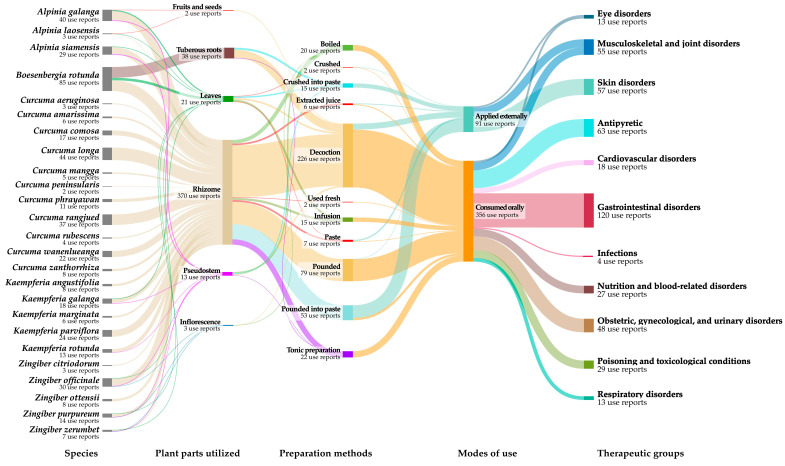
Sankey diagram of the relationships among Zingiberaceae species used for herbal medicine, plant parts utilized, preparation methods, modes of use, and therapeutic groups in Lop Buri Province, Thailand.

A total of 25 Zingiberaceae species were recorded for medicinal utilization during the present study (Figure 8). Among the recorded taxa, *Boesenbergia rotunda* showed the highest number of medicinal use reports (85 use reports), followed by *Curcuma longa* (44 use reports), *Alpinia galanga* (40 use reports), *C. rangjued* (37 use reports), *Zingiber officinale* (30 use reports), and *A. siamensis* (29 use reports).

Rhizomes were the most frequently utilized plant part, accounting for 370 use reports, followed by tuberous roots (38 use reports), leaves (21 use reports), pseudostems (13 use reports), inflorescences (3 use reports), and fruits and seeds (2 use reports). Rhizomes were prepared mainly by decoction (184 use reports), pounding (79 use reports), pounding into paste (53 use reports), tonic preparation (19 use reports), boiling (13 use reports), infusion (8 use reports), extracted juice (6 use reports), paste preparation (6 use reports), and use in fresh form (2 use reports). Tuberous roots were mainly prepared by decoction (29 use reports) and crushing into paste (7 use reports), whereas leaves were commonly prepared by infusion (7 use reports), decoction (6 use reports), and crushing into paste (5 use reports).

Decoction represented the most frequently recorded preparation method overall, accounting for 226 use reports, followed by pounding (79 use reports), pounding into paste (53 use reports), tonic preparation (22 use reports), boiling (20 use reports), infusion (15 use reports), crushing into paste (15 use reports), paste preparation (7 use reports), extracted juice (6 use reports), use in fresh form (2 use reports), and crushing (2 use reports).

Most medicinal preparations were administered through oral consumption (356 use reports), whereas external application accounted for 91 use reports. The medicinal applications were associated with 11 therapeutic groups. Gastrointestinal disorders showed the highest number of use reports (120 use reports), followed by antipyretic treatments (63 use reports), skin disorders (57 use reports), musculoskeletal and joint disorders (55 use reports), obstetric–gynecological and urinary disorders (48 use reports), poisoning and toxicology (29 use reports), nutrition and blood-related disorders (27 use reports), cardiovascular disorders (18 use reports), respiratory disorders (13 use reports), eye disorders (13 use reports), and infections (4 use reports).

#### 3.2.4. Used as Ornamental Plants

**Figure 9:**
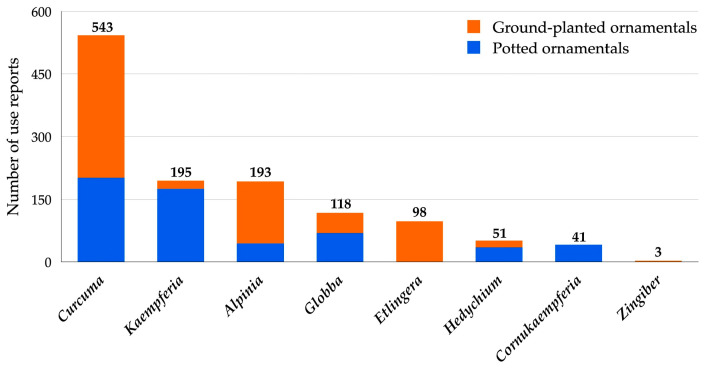
Ornamental utilization patterns of Zingiberaceae genera recorded in Lop Buri Province, Thailand, categorized into ground-planted ornamentals and potted ornamentals based on the number of use reports.

Ornamental utilization was recorded from eight genera of Zingiberaceae during the present study (Figure 9). *Curcuma* showed the highest number of ornamental use reports (543 use reports from 20 taxa), followed by *Kaempferia* (195 use reports from eight species), *Alpinia* (193 use reports from three species), *Globba* (118 use reports from ten species), *Etlingera* (98 use reports from one species), *Hedychium* (51 use reports from two species), *Cornukaempferia* (41 use reports from three species), and *Zingiber* (3 use reports from one species).

Ground-planted ornamentals accounted for most ornamental use reports in *Curcuma* (341 use reports), *Alpinia* (149 use reports), and *Etlingera* (98 use reports), whereas potted ornamentals predominated in *Kaempferia* (175 use reports), *Globba* (70 use reports), *Cornukaempferia* (41 use reports), and *Hedychium* (35 use reports). *Zingiber* was recorded only as ground-planted ornamentals with three use reports.

#### 3.2.5. Ritual Uses and Beliefs

**Figure 10:**
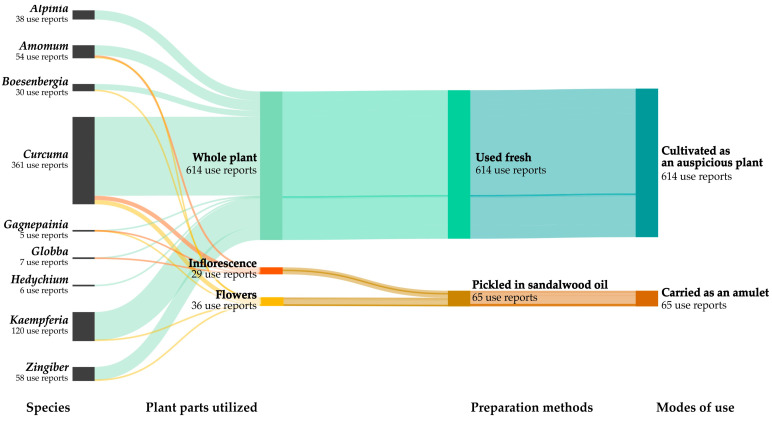
Ritual uses and beliefs associated with Zingiberaceae genera recorded in Lop Buri Province, Thailand, showing the relationships among utilized plant parts, preparation methods, and modes of use based on the number of use reports.

Ritual uses and beliefs associated with 64 Zingiberaceae species were recorded from nine genera during the present study (Figure 10). *Curcuma* showed the highest number of use reports (361 use reports from 33 species), followed by *Kaempferia* (120 use reports from 13 species), *Zingiber* (58 use reports from five species), *Amomum* (54 use reports from one species), *Alpinia* (38 use reports from two species), *Boesenbergia* (30 use reports from five species), *Globba* (7 use reports from three species), *Hedychium* (6 use reports from one species), and *Gagnepainia* (5 use reports from one species).

Whole plants represented the principal plant part utilized in ritual practices and beliefs, accounting for 614 use reports, followed by flowers (36 use reports) and inflorescences (29 use reports). Whole plants were mainly used fresh and cultivated as auspicious plants. Flowers and inflorescences were commonly pickled in sandalwood oil and carried as amulets, accounting for 65 use reports.

#### 3.2.6. Cosmetic Uses

**Figure 11:**
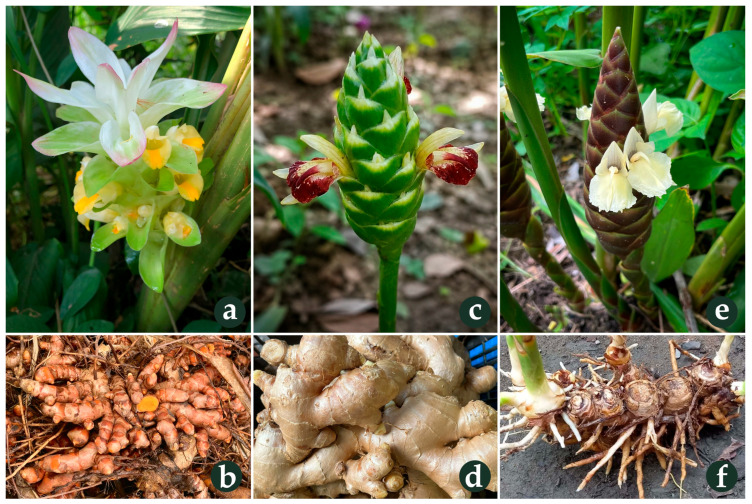
Zingiberaceae species used as cosmetic ingredients in Lop Buri Province, Thailand: (**a**,**b**) *Curcuma longa*; (**c**,**d**) *Zingiber officinale*; (**e**,**f**) *Zingiber purpureum*. Photographs by Thawatphong Boonma.

Cosmetic applications were recorded from three Zingiberaceae species during the present study (Figure 11). *Curcuma longa* showed the highest number of cosmetic use reports (22 use reports), followed by *Zingiber officinale* (16 use reports) and *Z. purpureum* (9 use reports). In all recorded cases, rhizomes were utilized and prepared by drying and grinding into powder before being used as ingredients in cosmetic products.

#### 3.2.7. Commercial Uses

**Figure 12:**
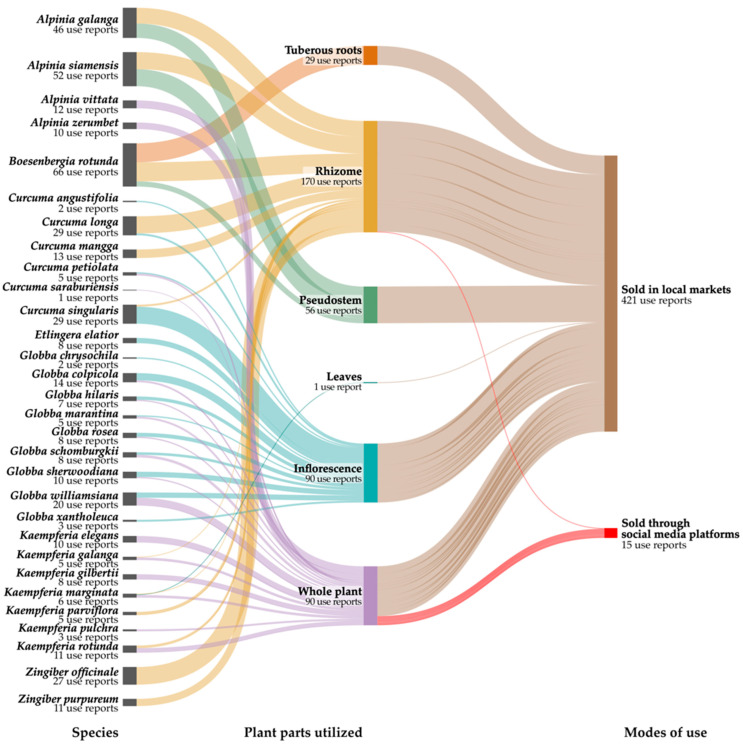
Commercial utilization patterns of Zingiberaceae species recorded in Lop Buri Province, Thailand, showing the relationships among utilized plant parts and modes of commercialization based on the number of use reports.

A total of 436 commercial use reports associated with 30 Zingiberaceae species were recorded during the present study (Figure 12). *Boesenbergia rotunda* showed the highest number of commercial use reports (66 use reports), followed by *Alpinia siamensis* (52 use reports), *A. galanga* (46 use reports), *Curcuma longa* and *C. singularis* (29 use reports each), *Zingiber officinale* (27 use reports), *Globba williamsiana* (20 use reports), *G. colpicola* (14 use reports), *C. mangga* (13 use reports), *A. vittata* and *Z. purpureum* (11 use reports each), and *G. sherwoodiana* and *Kaempferia elegans* (10 use reports each). Rhizomes represented the principal plant part involved in commercial utilization, accounting for 170 use reports, followed by inflorescences and whole plants (90 use reports each), pseudostems (56 use reports), tuberous roots (29 use reports), and leaves (1 use report). In all recorded cases, plant materials were commercialized in fresh form. Most commercialized plant materials were sold in local markets, accounting for 421 use reports, whereas only 15 use reports were associated with sales through social media platforms.

#### 3.2.8. Sources of Plant Materials and Sustainability Aspects

The sources of plant materials utilized by local communities in Lop Buri Province were closely associated with both cultivated and wild populations of Zingiberaceae (Figure 3). Cultivated taxa accounted for 104 taxa and were predominantly associated with home gardens, whereas 43 taxa were recorded from wild habitats, particularly limestone areas, dry evergreen forests, and mixed deciduous forests. Among the native taxa recorded during the present study, 43 taxa were recognized as endemic, many of which were associated with wild populations in natural habitats. In contrast, introduced taxa were represented exclusively by cultivated plants. These patterns indicate the important role of both cultivated and natural populations in supporting the ethnobotanical utilization of Zingiberaceae in Lop Buri Province.

#### 3.2.9. Seasonal Availability and Phenology

**Figure 13:**
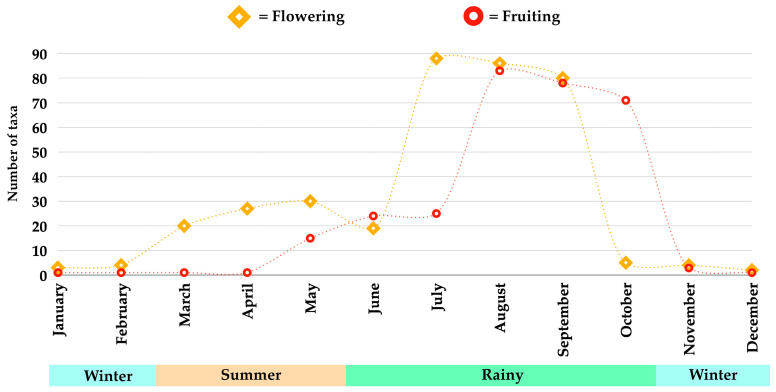
Monthly flowering and fruiting patterns of Zingiberaceae taxa recorded in Lop Buri Province, Thailand, across the winter, summer, and rainy seasons.

Flowering of Zingiberaceae taxa was recorded throughout the year, ranging from 2 to 88 taxa per month (Figure 13). The number of flowering taxa gradually increased from January (3 taxa) to May (30 taxa), decreased slightly in June (19 taxa), and reached a peak during the rainy season in July (88 taxa), followed by August (86 taxa) and September (80 taxa). Fruiting was also observed year-round, with 1–83 taxa recorded monthly. Fruiting taxa remained low from January to April (1 taxon each month), increased from May (15 taxa) to July (25 taxa), and peaked in August (83 taxa), followed by September (78 taxa) and October (71 taxa), before declining toward the winter season.

The predominance of cultivated taxa indicates that local ethnobotanical use is supported not only by wild plant resources but also by home gardens, agricultural areas, market exchange, and plant introduction from nearby areas. Introduced taxa were recorded exclusively under cultivation, and none were observed to establish self-sustaining populations or behave invasively in natural habitats during the present survey.

### 3.3. Quantitative Ethnobotanical Indices

#### 3.3.1. Species Use Value (SUV)

The SUV analysis revealed considerable variation in the ethnobotanical importance of Zingiberaceae taxa recorded in Lop Buri Province (Table 1). The highest SUVs were recorded for *Curcuma longa* (2.77), followed by *Zingiber officinale* (2.63), *Boesenbergia rotunda* (2.61), *Alpinia siamensis* (2.45), and *A. galanga* (2.30). These species were among the most frequently cited taxa by local informants and were associated with multiple utilization categories, particularly food, spice, medicinal, and commercial uses. Moderately high SUVs were also observed in several ornamental and culturally important taxa, e.g., *Curcuma mangga* (1.11), *Etlingera elatior* (1.04), and *C. singularis* (0.93). In contrast, many endemic or locally restricted taxa exhibited relatively low SUVs, reflecting their limited utilization or restricted availability in local communities. Species with high SUVs were generally characterized by broad utilization patterns, frequent cultivation, and widespread recognition among local informants, indicating their important role in the daily livelihoods, traditional knowledge systems, and local economies of Lop Buri Province.

#### 3.3.2. Genus Use Value (GUV)

**Figure 14:**
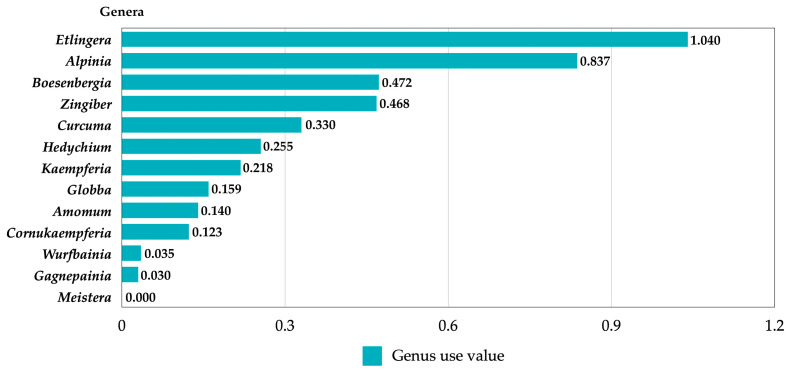
Genus use value (GUV) of Zingiberaceae recorded in Lop Buri Province, Thailand. Higher GUVs indicate greater relative ethnobotanical importance of each genus based on the cumulative species use values.

Genus Use Value (GUV) analysis demonstrated marked variation in the ethnobotanical importance among the recorded genera of Zingiberaceae in Lop Buri Province (Figure 14). *Etlingera* exhibited the highest GUV (1.040), followed by *Alpinia* (0.837), *Boesenbergia* (0.472), *Zingiber* (0.468), and *Curcuma* (0.330). Moderately high GUVs were also observed in *Hedychium* (0.255), *Kaempferia* (0.218), and *Globba* (0.159), whereas relatively low values were recorded in *Amomum* (0.140), *Cornukaempferia* (0.123), *Wurfbainia* (0.035), and *Gagnepainia* (0.030). In contrast, *Meistera* exhibited a GUV of 0.000 due to the absence of reported ethnobotanical uses among local informants.

#### 3.3.3. Relative Frequency of Citation

Relative Frequency of Citation (RFC) values varied considerably among the recorded Zingiberaceae taxa in Lop Buri Province (Table 1). The highest RFC values were recorded for *Alpinia siamensis*, *Boesenbergia rotunda*, *Curcuma longa*, and *Zingiber officinale* (1.000 each), indicating that these species were recognized and cited by all interviewed informants. Other species with relatively high RFC values included *A. galanga* (0.955), *Etlingera elatior* (0.891), *Kaempferia elegans* (0.555), and *C. singularis* (0.645). In contrast, several endemic or locally restricted taxa exhibited relatively low RFC values, particularly species known from limited populations or cultivated by only a few local households. Species without reported ethnobotanical uses, including *Amomum repoeense*, *Meistera koenigii*, *M. tomrey*, and *Wurfbainia uliginosa*, exhibited RFC values of 0.000.

#### 3.3.4. Plant Part Value (PPV)

Plant Part Value (PPV) analysis demonstrated that whole plants and rhizomes were the most important plant parts utilized in ethnobotanical practices associated with Zingiberaceae in Lop Buri Province (Figure 5). Whole plants exhibited the highest PPV (38.10%), followed closely by rhizomes (34.73%). Other commonly utilized plant parts included inflorescences (7.96%), pseudostems (7.92%), flowers (6.22%), and roots (3.46%). In contrast, leaves (1.25%), fruits and seeds (0.20%), and bracts (0.16%) showed relatively low PPVs.

#### 3.3.5. Informant Consensus Factor (Fic)

Informant Consensus Factor (Fic) values varied among the 11 therapeutic groups associated with medicinal uses of Zingiberaceae in Lop Buri Province (Table 2). The highest Fic value was recorded for infections (1.000), although this category was represented by only four use reports and one taxon. High Fic values were also observed for musculoskeletal and joint disorders (0.889), gastrointestinal disorders (0.849), antipyretic treatments (0.839), eye disorders (0.833), poisoning and toxicology (0.821), obstetric–gynecological and urinary disorders (0.809), and skin disorders (0.804). In contrast, relatively lower Fic values were recorded for cardiovascular disorders (0.706), respiratory disorders (0.667), and nutrition and blood-related disorders (0.654).

The present study revealed that numerous Zingiberaceae taxa in Lop Buri Province possess considerable ethnobotanical importance and are closely associated with local livelihoods, traditional medicine, food systems, ornamental horticulture, and cultural practices. However, several taxa were found to occur in restricted habitats, particularly limestone-associated ecosystems and remnant forest areas, which are increasingly threatened by habitat disturbance and land-use change. In addition, many endemic species were represented by small or localized populations observed during field surveys. These findings highlight the importance of conducting preliminary conservation assessments in order to support future monitoring, conservation planning, and sustainable utilization of Zingiberaceae diversity in Lop Buri Province.

### 3.4. Preliminary Conservation Status of Zingiberaceae in Lop Buri Province

The preliminary conservation assessment conducted in the present study focused primarily on taxa recorded from natural habitats and wild populations within Lop Buri Province (Table 1). Several endemic and habitat-restricted taxa associated with limestone ecosystems and remnant forest habitats were proposed to belong to threatened categories. Four species were proposed as Critically Endangered (CR): *Curcuma supraneeana*, *C. charanii*, *Kaempferia chaveerachiae*, and *K. saraburiensis*. Six species were proposed as Endangered (EN): *Curcuma saraburiensis*, *Globba colpicola*, *K. napavarniae*, *K. pardi*, *K. lopburiensis*, and *Zingiber brachystachys* (Figure 4). In contrast, many widespread or commonly cultivated taxa were assessed as Least Concern (LC), particularly species frequently cultivated in home gardens and agricultural systems, including *Alpinia galanga*, *Boesenbergia rotunda*, *C. angustifolia*, *K. rotunda*, and *Z. parishii*. In addition, *C. × lopburiensis* was provisionally assessed as Data Deficient (DD) due to its putative hybrid origin and limited available ecological and population data.

Field observations indicated that habitat degradation, forest disturbance, agricultural expansion, tourism development, and wild harvesting may represent important threats affecting several native populations, particularly taxa restricted to limestone habitats and fragmented forest areas. Although cultivation appears to reduce harvesting pressure on some economically important species, several endemic taxa remained represented by relatively small or localized wild populations observed during field surveys. These findings highlight the importance of continued field monitoring, habitat protection, ex situ conservation, and sustainable utilization strategies for Zingiberaceae diversity in Lop Buri Province.

Although several introduced Zingiberaceae taxa were recorded in the present study, none were observed to behave invasively or to establish self-sustaining populations in natural habitats. Most introduced taxa were cultivated in home gardens, agricultural areas, or ornamental collections. In contrast, several native and endemic taxa were rare, locally restricted, or associated with limestone habitats, and therefore require conservation attention.

### 3.5. A Putative Natural Hybrid in Curcuma

#### 3.5.1. Field Observations

The putative natural hybrid was discovered in limestone-associated dry evergreen forest in the Khok Samrong District, Lopburi Province, central Thailand, where the putative parental taxa, *Curcuma saraburiensis* and *C. parviflora*, occur sympatrically or near-sympatrically within the same limestone-associated forest. Individuals of both putative parental species were observed within approximately 5–15 m of the putative hybrid population, with *C. saraburiensis* occurring in closer proximity and being more abundant in the immediate surrounding area, whereas *C. parviflora* was found slightly farther away within the same forest. The putative hybrid population was small and localized, consisting of five flowering individuals observed during the field survey. All taxa were observed to flower simultaneously during the same season, indicating overlapping reproductive periods that may facilitate natural hybridization.

Morphologically, the putative hybrid exhibited a combination of characters observed in both putative parental taxa. The reddish markings on the labellum resembled those of *C. saraburiensis*, whereas the purple and undulate distal portion of the labellum was similar to *C. parviflora*. The coma bracts varied from pale green to white with green margins or green apices, corresponding to conditions observed in the putative parental taxa, whereas the fertile bracts were either patterned similarly to those of *C. saraburiensis* or plain green as in *C. parviflora*. Although the overall morphology tended to more closely resemble *C. saraburiensis*, several floral characters remained intermediate and variable among individuals, which is inconsistent with the typical morphology observed in natural populations of either parental species. No mature fruits were observed during the study period. Taken together, the sympatric or near-sympatric occurrence of the putative parental taxa, overlapping flowering periods, localized occurrence, and intermediate morphology collectively support the interpretation of these plants as a putative natural hybrid, which is herein described as *Curcuma × lopburiensis* nothosp. nov.

#### 3.5.2. Morphological Comparisons

**Figure 15:**
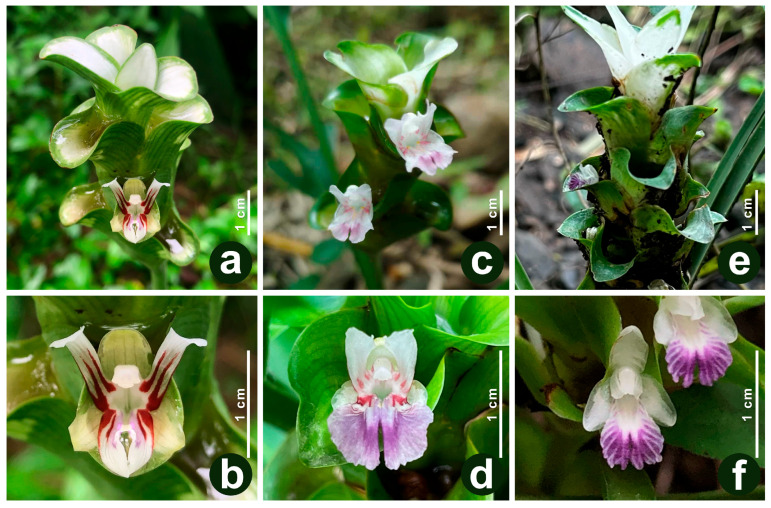
Comparative floral morphology of putative parental taxa and the proposed natural hybrid. (**a**,**b**) *Curcuma saraburiensis*; (**c**,**d**) *C. × lopburiensis*; (**e**,**f**) *C. parviflora*. Photographs by Thawatphong Boonma.

*Curcuma × lopburiensis* exhibits a combination of character states observed in *C. saraburiensis* and *C. parviflora* (Figure 15, Table 3). The putative hybrid shares several vegetative and floral traits with *C. saraburiensis*, including general habit, flower size, inflorescence structure, number of flowers per fertile bracts, and labellum size, whereas the purple distal portion and slightly crisped margins of the labellum are more similar to those of *C. parviflora*. In addition, several characters in *C. × lopburiensis*, including fertile bract coloration, coma bract morphology, calyx length, and lower leaf surface indumentum, exhibit intermediate or variable conditions between the two putative parental taxa.

#### 3.5.3. Morphometric Analysis

**Figure 16:**
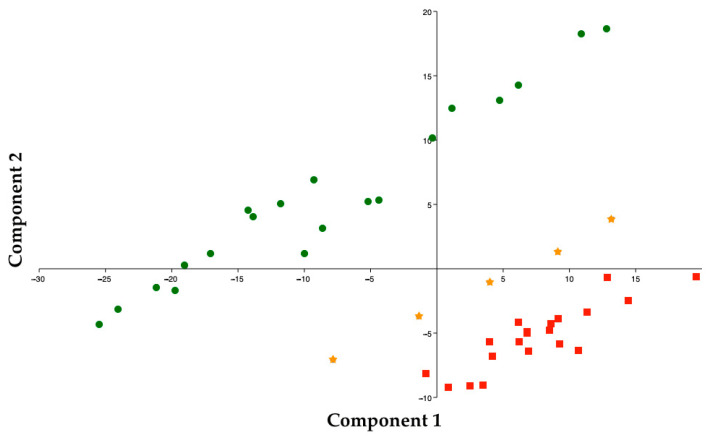
Principal Component Analysis (PCA) scatter plot based on 22 quantitative morphological characters of *Curcuma saraburiensis* (red squares), *C. × lopburiensis* (yellow stars), and *C. parviflora* (green circles).

Principal Component Analysis (PCA) based on 22 quantitative vegetative and floral characters separated the three taxa into distinct morphological groups (Figure 16, Table A1). The ordination pattern demonstrated that *Curcuma saraburiensis* (red squares) and *C. parviflora* (green circles) formed two well-defined clusters with no substantial overlap, whereas *C. × lopburiensis* (yellow stars) occupied an intermediate position between the two taxa. This intermediate placement is consistent with the hypothesis that *C. × lopburiensis* originated through natural hybridization between *C. saraburiensis* and *C. parviflora*.

The first principal component (PC1) primarily reflected variation in vegetative morphology, particularly leafy shoot height, leaf blade dimensions, petiole length, and peduncle length. Specimens of *C. parviflora* were mainly distributed on the negative side of PC1 and were characterized by generally smaller vegetative organs, narrower leaves, shorter inflorescences, and reduced floral dimensions. In contrast, *C. saraburiensis* clustered predominantly on the positive side of PC1, corresponding to its generally more robust habit, broader leaves, larger inflorescences, and larger floral organs. Individuals of *C. × lopburiensis* occupied positions between the two parental taxa and exhibited intermediate values for many characters, especially leaf size, peduncle length, fertile bract dimensions, and floral measurements.

The second principal component (PC2) was associated mainly with floral characters, including calyx length, labellum size, lateral staminode dimensions, and fertile bract morphology. Although some intraspecific variation was observed within both parental taxa, the three groups remained relatively distinct in the ordination space. The placement of *C. × lopburiensis* between *C. saraburiensis* and *C. parviflora* is consistent with the intermediate morphology observed in the field and further supports its interpretation as a putative natural hybrid. Nevertheless, additional evidence is needed to confirm its parentage and the direction of hybridization.

#### 3.5.4. Taxonomic Treatment

***Curcuma × lopburiensis* Boonma, Saensouk & P.Saensouk, nothosp. nov.**

**Type:** THAILAND, Central, Lop Buri Province, Khok Samrong District, c. 150 m a.s.l., 24 September 2025, *T.Boonma LB035* (Holotype VMSU: Including flowers preserved in spirit as part of a single specimen).

**Figure 17:**
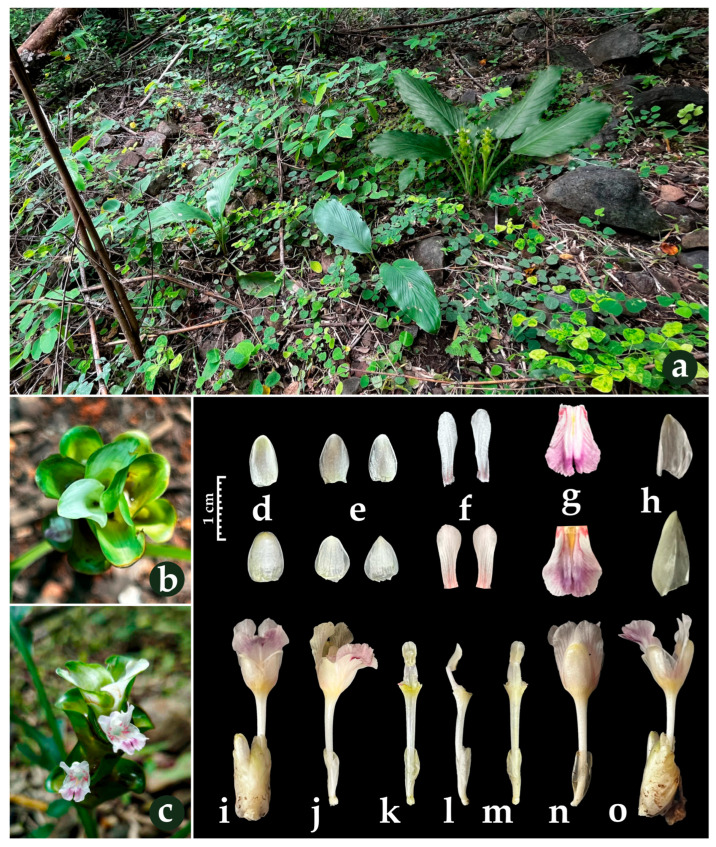
*Curcuma × lopburiensis* Boonma, Saensouk & P.Saensouk, nothosp. nov.: (**a**) habit in limestone-associated forest; (**b**) top view of inflorescence; (**c**) side view of inflorescence; (**d**–**g**) intraspecific variation in floral parts: (**d**) dorsal corolla lobes; (**e**) lateral corolla lobes; (**f**) lateral staminodes; (**g**) labella; (**h**) bracteole; (**i**,**o**) flower and buds in a cincinnus, shown in different views; (**j**) flower, side view; (**k**–**m**) dissected flower showing anther, floral tube, calyx, and ovary in front, side, and back views; (**n**) flower, back view. Photographs by Thawatphong Boonma.

**Figure 18:**
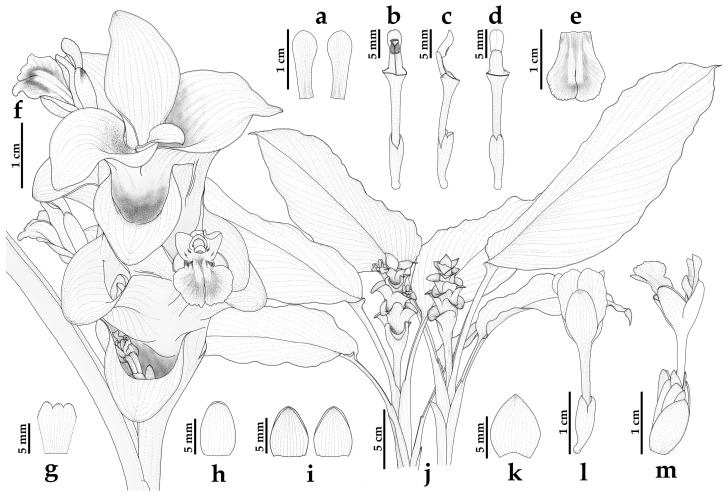
*Curcuma × lopburiensis* Boonma, Saensouk & P.Saensouk, nothosp. nov.: (**a**) lateral staminodes; (**b**–**d**) dissected flower showing anther, floral tube, calyx, and ovary, in front, side, and back views; (**e**) labellum; (**f**) inflorescence with flowers; (**g**) dissected calyx; (**h**) dorsal corolla lobe; (**i**) lateral corolla lobes; (**j**) habit with inflorescences; (**k**) bracteole; (**l**) flower, back view; (**m**) flower and buds in a cincinnus. Drawn by Thawatphong Boonma.

**Diagnosis:** *Curcuma × lopburiensis* differs from *C. parviflora* by its taller leafy shoots, longer petioles, larger inflorescences, fertile bracts with more flowers, larger flowers, longer calyx, and larger labellum with a more deeply incised apex. It differs from *C. saraburiensis* by its shorter inflorescences and calyx, fewer-flowered cincinni, smaller flowers, and labellum with a purple distal part and slightly crisped margins resembling *C. parviflora*. The putative hybrid is intermediate between the two parental species in several vegetative and floral characters, particularly in calyx length, inflorescence size, fertile bract morphology, and labellum coloration and shape (Table 3, Figure 15, Figure 17 and Figure 18).

**Description:** Perennial herb with a short ovoid rhizome, cream to pale buff internally and mildly fragrant, 2–3 × 1–1.5 cm. Root tubers ovoid to ellipsoid with fibrous roots. Leafy shoot 30–50 cm tall; bladeless sheaths 1–2, green, with reddish tinge at base, or red. Leaf sheaths green or with red at base; ligule bilobed, 2–3 mm long; petiole canaliculate, green, 4–12 cm. Leaves 3–5, elliptic to narrowly ovate, 13–26 × 5.5–12 cm wide, apex acuminate, base cuneate–oblique to rounded, margin entire, slightly undulate, green on both surfaces, adaxial surface glabrous; abaxial surface glabrous or puberulent. Inflorescence terminal on the pseudostem; thyrse cylindrical, 7–9 cm long; peduncle green, 10–20 cm long, glabrous. Fertile bracts 10–14 in number, containing 4–6 flowers in each bract, obovate to broadly obovate, apex obtuse to rounded, recurved distal part, green, 2–3 × 1–2.5 cm. Coma bracts few, ovate to broadly ovate, glabrous, outer side green, inner side pale green or white with green edges, or white with green apex, glabrous, 2–3 × 1–2 cm, apex obtuse. Bracteoles whitish, ovate, 6–9 × 6–7 mm, acute apex. Flowers 3–3.2 cm long. Calyx whitish, tubular, 8–10 mm long, apex trilobed, glabrous. Floral tube white, c. 2 cm long, glabrous; dorsal corolla lobe, ovate, 8–9 × 4–5 mm, light pale yellow, glabrous; lateral corolla lobes, ovate, 7–8 × 4–5 mm, light pale yellow, glabrous. Lateral staminodes narrowly oblong, (10–)12–13 × 3–4 mm, rounded to obtuse apex, white to very light pale purple, each with 2–3 pale reddish lines at base. Labellum obovate, 11–13 × 8–10 mm, deeply bilobed with an incision up to 5 mm, white centrally, purple distally and on either side, with short pale reddish lines on each side near the base, mid lobe with or without yellowish patch at the basal–central part, margins slightly crisped. Stamen 1; filament flat, white or with reddish tinge; anther 4–5 mm long, spurs absent; crest 1.5 × 1.5 mm, apex rounded, sometimes slightly retuse. Ovary subspherical, 1–2 × 1.5–2 mm, tricarpellate, glabrous. Style very slender. Stigma white c. 1 × 1 mm, glabrous. Epigynous glands absent. Fruit and seeds not seen.

***Etymology:*** The epithet “*lopburiensis*” refers to Lop Buri Province in central Thailand, where the putative natural hybrid was discovered and is currently known to occur.

***Thai Vernacular name:*** “*Sri Chantra*”, named in honor of Sirima Boonsaeng, whose assistance during field exploration contributed to the discovery of this putative natural hybrid. The name is formed from two elements: *Sri*, derived from *Siri* in her given name, and *Chantra*, referring to Khao Wong Phrachan, a well-known mountain near the discovery site in Lop Buri Province and a place associated with local legend.

***Distribution:*** *Curcuma × lopburiensis* is currently known only from Lop Buri Province, central Thailand, where it occurs sympatrically with its putative parental species, *C. saraburiensis* and *C. parviflora*.

***Ecology:*** It grows in deciduous to dry evergreen forest and semi-open areas associated with limestone areas on sandy to rocky soils.

***Phenology:*** Flowering occurs during the rainy season from June to September, and dormancy begins in November.

***Notes:*** The fertile bracts of *Curcuma × lopburiensis* are variable in coloration, being either plain green or marked with pale longitudinal lines and white patches as in *C. saraburiensis*. Likewise, the coma bracts vary from plain pale green to forms resembling those of either *C. saraburiensis* or *C. parviflora*. Although *C. × lopburiensis* is here interpreted as a putative natural hybrid most likely involving *C. saraburiensis* and *C. parviflora*, its flower may superficially resemble that of *C. charanii* when viewed from the front, particularly in the purple coloration of the labellum and the relatively broad lateral staminodes. However, the yellowish marking in *C. × lopburiensis* is not clearly differentiated into two distinct spots as in *C. charanii*. The putative hybrid also differs from *C. charanii* by its well-developed coma bracts, fewer flowers per fertile bract, longer calyx, and different overall inflorescence morphology. In addition, *C. charanii* was not observed in the immediate vicinity of the hybrid population during the present survey, whereas *C. saraburiensis* and *C. parviflora* occurred nearby and flowered simultaneously. Therefore, *C. parviflora* is considered the more plausible second parental species, although molecular evidence is needed to confirm the parentage and direction of hybridization.

**Additional specimen examined:** Thailand, Central, Lop Buri Province, Khok Samrong District, c. 155 m a.s.l., 24 September 2025, *T.Boonma LB111* (VMSU).

## 4. Discussion

The present study substantially expands the known diversity of Zingiberaceae in Lop Buri Province, increasing the provincial record from 14 previously documented [10] species to 110 taxa, including 109 species and one putative natural hybrid. This remarkable increase demonstrates that the diversity of Zingiberaceae in central Thailand has previously been underestimated, particularly in provinces where systematic field surveys have been limited. The high number of newly recorded taxa for Lop Buri Province also emphasizes the importance of repeated field exploration throughout different seasons, as many species of Zingiberaceae are seasonal, short-flowering, or restricted to particular microhabitats and may therefore be overlooked during general botanical surveys [10,15]. These findings support the expectation of the present study that the previously known diversity of Zingiberaceae in Lop Buri Province did not fully reflect the actual diversity of the family in the area.

The composition of Zingiberaceae in Lop Buri Province reflects both the general diversity pattern of the family in Thailand and the ecological heterogeneity of the province. The dominance of *Curcuma*, followed by *Kaempferia*, *Globba*, and *Zingiber*, is consistent with the high diversity of these genera in the Thai flora [10]. However, the number of taxa recorded in Lop Buri is noteworthy because the province belongs to the Central Floristic Region, which has received less attention than northern, northeastern, or peninsular Thailand in studies of Zingiberaceae [37,38,39]. The present findings therefore demonstrate that central Thailand remains important for floristic exploration, especially in areas where limestone formations, forest fragments, agricultural landscapes, and home gardens occur in close proximity.

Habitat heterogeneity appears to be a major factor contributing to the high diversity recorded in the present study. Lop Buri Province contains dry evergreen forest, mixed deciduous forest, limestone-associated habitats, agricultural areas, and home gardens, each supporting different components of Zingiberaceae diversity. Natural habitats were especially important for native and endemic taxa, whereas cultivated taxa were mainly concentrated in home gardens and agricultural areas. Limestone-associated habitats supported a particularly important component of the flora, including several endemic and habitat-restricted species. This pattern is consistent with the expectation that natural habitats, particularly limestone-associated areas, support important native and endemic taxa in Lop Buri Province. The occurrence of numerous wild and endemic taxa in limestone areas, mixed deciduous forest, and dry evergreen forest further highlights the conservation value of these habitats [3,15,16,19].

The difference between previous records and the present findings should be interpreted in relation to the survey scale and data scope. The Flora of Thailand [10] provides national taxonomic treatments and distribution records based on available specimens and documented collections, but it does not necessarily represent a complete inventory of all subprovincial habitats. In the present study, repeated field surveys across seasons and habitat types revealed 43 wild taxa in natural habitats, including several species previously known from nearby provinces but not formally documented from Lop Buri Province. This pattern suggests that the wild Zingiberaceae diversity of Lop Buri Province had been underestimated, particularly in limestone-associated and remnant forest habitats.

**Figure 19:**
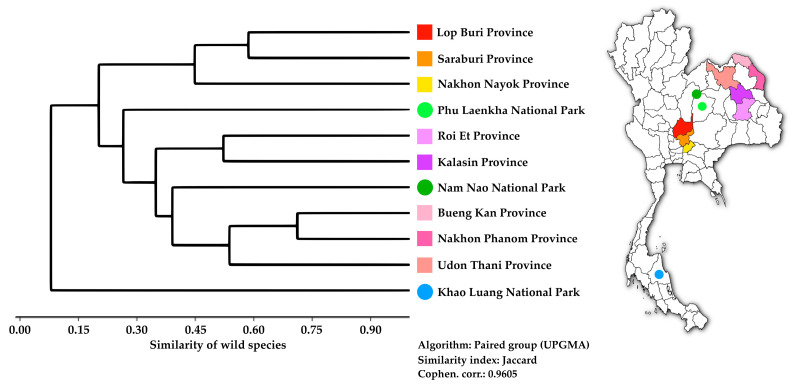
Comparative analysis of wild Zingiberaceae species recorded in Lop Buri Province and selected areas previously studied in Thailand [3,15,28,38,39,40,41,42,43,44].

The comparative similarity analysis of wild Zingiberaceae species places the Lop Buri flora within a broader regional context (Figure 19). Based on Jaccard similarity and UPGMA clustering, the wild species composition of Lop Buri corresponds most closely to that of Saraburi, and this pair is subsequently grouped with Nakhon Nayok. This pattern suggests that Lop Buri shares stronger floristic similarity with nearby central Thai areas than with more distant regions. The close relationship between Lop Buri and Saraburi likely reflects their geographical proximity, similar habitat conditions, and the presence of limestone-associated habitats that support several native or endemic taxa occurring in central Thailand [15,19]. The subsequent association with Nakhon Nayok may reflect additional shared forest-associated elements among central Thai provinces with heterogeneous landscapes, forest fragments, and foothill habitats [3]. This floristic connection also helps explain the occurrence in Lop Buri Province of several taxa previously reported from nearby central Thai areas. For example, some limestone-associated taxa originally described or reported from Saraburi and adjacent regions were also confirmed in Lop Buri during the present field surveys. These findings emphasize that provincial boundaries do not necessarily correspond to biological distribution limits, especially for taxa associated with continuous or closely related limestone systems.

In contrast, northeastern localities and protected areas form separate clusters, including Roi Et with Kalasin and Bueng Kan with Nakhon Phanom, while Udon Thani is positioned close to the latter group. Phu Laenkha National Park and Nam Nao National Park are more distant from the Lop Buri–Saraburi–Nakhon Nayok cluster, and Khao Luang National Park is clearly separated from all other areas. These patterns probably reflect differences in floristic region, geography, elevation, climate, vegetation type, and habitat composition among the compared areas [3,15,28,38,39,40,41,42,43,44]. Because the analysis is based only on wild species, the clustering pattern is most appropriately interpreted as similarity in natural species composition rather than overall ethnobotanical or cultivated diversity. Moreover, comparisons among areas should be interpreted with caution because differences in sampling intensity, survey duration, habitat coverage, and taxonomic scope may influence the number of taxa recorded in each study [10,15].

The occurrence of many cultivated taxa also reflects the close relationship between Zingiberaceae and local livelihoods. Home gardens serve as important reservoirs of useful Zingiberaceae, including food, spice, medicinal, ornamental, ritual uses and beliefs, and commercial species. These cultivated assemblages contribute to household plant diversity and may help maintain traditional knowledge [3,4,5,6,7,12,13,14,15,17]. However, cultivated diversity must be interpreted separately from wild diversity. Some taxa recorded in cultivation may reflect plant exchange, market availability, horticultural preference, or introduction from outside the province rather than natural occurrence. Introduced and cultivated taxa contributed substantially to ethnobotanical diversity, but they should not be interpreted in the same way as naturally occurring taxa. Their presence reflects human-mediated movement, household cultivation, and market exchange, whereas wild taxa better represent the natural floristic composition of the province. Distinguishing among wild, cultivated, and wild/cultivated taxa is therefore essential for interpreting floristic diversity, ethnobotanical importance, and conservation value.

The ethnobotanical findings show that Zingiberaceae in Lop Buri Province are not merely components of plant diversity, but are also integrated into everyday life, household practices, cultural traditions, and local economies. The wide range of recorded uses indicates that the family has a multifunctional role in local communities. Rather than being valued only as medicinal plants, members of Zingiberaceae are used in food culture, spice preparation, ornamental cultivation, ritual practices, cosmetics, and commerce [3,15,28,38,40,41,42,43,44]. This multifunctionality helps explain why many species are actively maintained in home gardens and cultivated landscapes, even when they are not naturally distributed in the immediate surroundings.

The most frequently cited species were generally widespread, easily cultivated, and familiar to local people. Their high ethnobotanical importance is likely associated with accessibility, cultural familiarity, and multiple practical uses [3,15,28,38,40,41,42,43,44]. In contrast, several endemic or habitat-restricted taxa showed low use values. This does not necessarily indicate low cultural or biological importance, but may reflect limited availability, narrow distribution, small population size, or reduced interaction with local communities. Therefore, species with high use values and species with high conservation concern represent different but complementary priorities. Common cultivated species are important for sustaining local livelihoods and traditional practices, whereas narrowly distributed wild species require habitat protection and population monitoring. This distinction is important because conservation planning based only on use frequency may overlook rare or endemic species with limited local use but high biological significance.

The use of rhizomes and whole plants has different implications depending on the source of plant material. When these parts are obtained from cultivation, harvesting pressure on natural populations may be limited. However, if rhizomes or whole plants are collected from small wild populations, especially in limestone-associated habitats, such practices may contribute to population decline. This is particularly relevant for endemic or localized species occurring in fragmented habitats. Sustainable use strategies should therefore encourage cultivation, propagation, and local awareness while discouraging destructive harvesting from natural populations [45,46].

The ethnobotanical patterns documented in this study also suggest that traditional knowledge is dynamic and influenced by contemporary social and economic conditions [46,47]. Food and spice uses remain closely connected with daily life and local cuisine, whereas medicinal uses may be affected by increasing access to modern healthcare and changes in intergenerational knowledge transmission [48,49,50,51]. Some plant uses associated with older cultural practices may also become less frequent as younger generations rely less on these traditions or no longer encounter the same contexts in which such knowledge was formerly applied. Consequently, certain aspects of local ethnobotanical knowledge may gradually erode over time if they are not actively practiced, transmitted, or documented [46,47,48,49,50,51]. Ornamental, ritual, and commercial uses further show that the value of Zingiberaceae extends beyond subsistence and medicine to include aesthetics, belief systems, cultural identity, and income generation [3,4,13]. These findings emphasize the need to consider ethnobotanical knowledge as a living and changing system rather than a fixed inventory of plant uses.

The preliminary conservation assessment highlights the importance of Lop Buri Province for the conservation of endemic and habitat-restricted Zingiberaceae. Several taxa are proposed here as threatened at the regional level because they occur in small populations, have limited local distributions, or are associated with habitats affected by disturbance. Limestone-associated taxa deserve particular attention because limestone hills are often spatially isolated and vulnerable to quarrying, land-use conversion, tourism development, fire, invasive species, and habitat fragmentation [15]. The conservation statuses proposed in this study should be regarded as preliminary regional assessments because they are based on field observations within Lop Buri Province, available distributional information, population estimates, and observed threats, following IUCN Red List criteria [35,36]. Nevertheless, these assessments are useful for identifying local conservation priorities and guiding future surveys. Taxa for which no conservation status is proposed were mainly cultivated or not assessed because the present study focused on conservation evaluation of naturally occurring populations in natural habitats of Lop Buri Province.

The preliminary conservation assessments presented here are intended to support conservation planning rather than to promote exploitation. Ethnobotanical information represents existing local knowledge and should not be interpreted as encouragement for further wild harvesting, especially for threatened taxa. The absence of precise locality coordinates in the manuscript provides an additional safeguard for rare and threatened species. These data may be useful for relevant conservation agencies in Thailand for identifying priority habitats, particularly limestone-associated sites, and may support area-based conservation approaches, including Other Effective Area-Based Conservation Measures (OECMs), population monitoring, habitat protection, ex situ cultivation, and community-based awareness programs.

The discovery of *Curcuma × lopburiensis* nothosp. nov. provides additional evidence that limestone-associated habitats in central Thailand may support not only high species diversity but also ongoing evolutionary processes [15,52,53,54,55,56]. The putative hybrid was found in a small and localized population in limestone-associated dry evergreen forest, where *C. saraburiensis* and *C. parviflora* occurred sympatrically or near-sympatrically and flowered during the same period [10,15,57]. Its morphology combines characters of both putative parental taxa, including variation in fertile bract and coma bract coloration, labellum coloration, and floral form. The reddish markings of the labellum resemble *C. saraburiensis*, whereas the purple and slightly undulate distal portion of the labellum is similar to *C. parviflora* [10]. These observations, together with the localized occurrence and absence of mature fruits observed during the study period, support the interpretation of this taxon as a putative natural hybrid rather than a fully independent species.

Morphometric evidence further supports the distinctiveness of *Curcuma × lopburiensis* from the two putative parental taxa. The PCA results indicate that individuals of the putative hybrid occupy an intermediate or partially overlapping morphospace between *C. saraburiensis* and *C. parviflora*, while retaining several features closer to *C. saraburiensis*. This pattern is consistent with field observations that the putative hybrid resembles *C. saraburiensis* in general habit and inflorescence structure but differs in several floral characters. However, the small number of hybrid individuals limits statistical interpretation, and qualitative characters such as floral coloration, bract pattern, and indumentum remain important for recognizing the hybrid condition [58,59,60,61,62].

Documenting *Curcuma × lopburiensis* as a putative natural hybrid is important because it establishes a clear record of its natural occurrence, habitat, and association with the putative parental species. Similar field-based documentation has been used in the recognition of natural hybrids and putative hybrids in other plant groups, including Zingiberaceae and Orchidaceae [58,62]. In groups such as Zingiberaceae, morphologically distinctive plants may enter cultivation or be exchanged among growers, and their original provenance can become unclear over time, as shown in the case of horticulturally introduced hybrid bamboo in Southeast Asia [61]. Without field-based documentation, such plants may later be difficult to interpret taxonomically or conservationally. The present treatment therefore provides a baseline for recognizing the population as a naturally occurring, morphologically intermediate entity, while avoiding premature recognition as an independent species. This approach also supports a more appropriate conservation interpretation and provides a foundation for future molecular confirmation [60].

Future molecular studies would provide an important additional test of the hybrid origin of *Curcuma × lopburiensis* [60]. Population-level sampling using both plastid and nuclear markers, or genome-wide data, would help evaluate its relationship with the putative parental species and clarify whether it represents an F1 hybrid, a later-generation hybrid, a backcross, or a stabilized hybrid lineage [60,61]. Recognition as a putative natural hybrid is therefore a cautious treatment that reflects its distinct morphology, field occurrence, and ecological context without overinterpreting the precise direction or generation of hybridization [58,62].

The case of *Curcuma × lopburiensis* also has broader taxonomic implications for *Curcuma* subgenus *Hitcheniopsis*. Natural hybridization may complicate species delimitation, particularly when closely related taxa occur sympatrically, flower simultaneously, and produce morphologically intermediate individuals [58,60,62]. In such cases, reliance on a single morphological character may lead to misidentification, especially when floral coloration and bract morphology are variable or intermediate. The present study therefore demonstrates the importance of combining field ecology, population context, detailed morphology, morphometrics, and comparison with sympatric species when evaluating morphologically intermediate populations.

Although the present study provides the most comprehensive account of Zingiberaceae in Lop Buri Province to date, some limitations should be acknowledged. Field surveys were conducted during 2024–2025, and additional seasonal or long-term observations may reveal further taxa, especially species with short flowering periods. Ethnobotanical information was based on interviews with local informants and therefore reflects knowledge available during the study period. In addition, the conservation assessments proposed here are preliminary regional evaluations, and the hybrid origin of *Curcuma × lopburiensis* requires molecular confirmation.

The present study demonstrates that Lop Buri Province is an important but previously underdocumented area for Zingiberaceae diversity, ethnobotanical knowledge, and conservation in central Thailand. The province supports a rich assemblage of native, endemic, cultivated, and introduced taxa, with natural habitats—especially limestone-associated systems—playing a key role in maintaining wild and endemic diversity. Ethnobotanical findings reveal that Zingiberaceae remain important in local food systems, traditional knowledge, ornamental horticulture, ritual practices, and local economies. At the same time, the discovery of *Curcuma × lopburiensis* highlights the evolutionary significance of habitats where closely related species occur together. Future studies should expand floristic comparisons across additional provinces in central Thailand, apply molecular approaches to confirm the origin of *C. × lopburiensis*, monitor small populations of endemic and limestone-associated taxa, and evaluate how local ethnobotanical knowledge of Zingiberaceae changes across generations and under ongoing socio-economic transformation.

## 5. Conclusions

This study provides the first comprehensive account of the diversity, ethnobotanical uses, preliminary regional conservation status, and selected taxonomic notes of Zingiberaceae in Lop Buri Province, central Thailand. A total of 110 taxa, comprising 109 species and one putative natural hybrid, were recorded from the province, representing 13 genera and three tribes. Among these, 43 taxa were recorded from natural habitats as wild or naturally occurring populations, while the total of 110 taxa also included cultivated, introduced, and locally utilized taxa. The results substantially increase the known diversity of Zingiberaceae in Lop Buri, with 95 taxa newly recorded for the province, and demonstrate that the family has been considerably underestimated in this region. Natural habitats, especially limestone-associated areas, mixed deciduous forests, and dry evergreen forests, were shown to support important native and endemic taxa, highlighting the conservation value of habitat heterogeneity in central Thailand.

The ethnobotanical survey documented extensive local knowledge associated with Zingiberaceae, with 106 taxa reported to have uses in food, spice, medicinal, ornamental, ritual and belief, cosmetic, and commercial categories. Food-related uses were the most frequently recorded, followed by ornamental, spice, ritual, medicinal, commercial, and cosmetic uses. These findings indicate that Zingiberaceae remain closely integrated in local livelihoods, household practices, cultural traditions, and small-scale economic activities. However, the uneven distribution of knowledge among use categories suggests that some forms of traditional knowledge, particularly those related to medicine and ritual practices, may be vulnerable to gradual decline under changing social and economic conditions.

The preliminary conservation assessment further emphasizes the importance of natural habitats in Lop Buri Province, particularly for endemic species and taxa with restricted distributions. Several wild populations occur in localized or disturbed habitats and may be affected by habitat degradation, land-use change, and limited population size. The putative natural hybrid, here described as *Curcuma × lopburiensis*, also underlines the taxonomic and evolutionary significance of sympatric populations within the genus *Curcuma*. This study contributes baseline data for floristic research, ethnobotanical documentation, conservation planning, and future taxonomic studies of Zingiberaceae in Thailand. Continued field surveys, population monitoring, and molecular investigations are recommended to clarify taxonomic relationships, confirm hybrid origin, and support the long-term conservation of native and endemic Zingiberaceae in the region.

## Figures and Tables

**Table 1:** Diversity of Zingiberaceae taxa recorded in Lop Buri Province, Thailand, including vernacular names, distribution status, occurrence types, habitats, phenology, utilization, species use value (SUV), relative frequency of citation (RFC), conservation status, and voucher specimens.

No.	Scientific Name	Vernacular Name	Distribution Status for Thailand	Occurrence Type ^1^	Habitats ^2^	Phenology ^3^		Utilization		SUV	RFC	Conservation Status ^6^		Voucher Specimens
						Flowering	Fruiting	Used Parts ^4^	Purposes ^5^			IUCN	Author Proposed	
1.	*Alpinia galanga* (L.) Willd.	Kha	Native	W/C	AG, DE, HG, MX	4–9	6–10	Fs, Fw, In, Ls, Ps, Rz	CC, FD, SP, MC	2.30	0.955	NE	LC	Boonma LB001
2	*Alpinia laosensis* Gagnep.	Kha Dok Daeng	Native	W/C	DE, HG	5–8	6–10	Fs, Fw, In, Ls, Ps, Rz	FD, SP, MC	0.14	0.073	NE	LC	Boonma LB002
3	*Alpinia macroura* K.Schum.	Kha Pa	Native	W	DE	3–6	5–10	Ps	FD	0.02	0.018	NE	LC	Boonma LB003
4	*Alpinia mutica* Roxb.	Kha Nam	Native	C	HG	3–6	5–10	Wp	RT	0.25	0.255	NA	NA	Boonma LB004
5	*Alpinia oxymitra* K.Schum.	Laow	Native	W	DE	2–5	5–9	Fw, In, Ps	FD	0.33	0.327	NE	LC	Boonma LB005
6	*Alpinia purpurata* (Vieill.) K.Schum.	Kha Daeng	Introduced	C	AG, HG	1–12	Not seen	Wp	OM	0.76	0.764	NA	NA	Boonma LB006
7	*Alpinia siamensis* K.Schum.	Kha Ta Daeng	Native	C	AG, HG	5–8	5–11	Fw, In, Ls, Ps, Rz	CC, FD, SP, MC	2.45	1.000	NA	NA	Boonma LB007
8	*Alpinia vittata* W.Bull	Kha Daang	Introduced	C	AG, HG	6–8	7–9	Wp	CC, OM, RT	0.90	0.700	NA	NA	Boonma LB008
9	*Alpinia zerumbet* (Pers.) B.L.Burtt & R.M.Sm.	Kha Daang Lueang	Native	C	AG, HG	3–6	6–8	Wp	CC, OM	0.38	0.291	NA	NA	Boonma LB009
10	*Amomum foetidum* Boonma & Saensouk	Ton Maeng Kaeng	Endemic	C	HG	1–3	Not seen	Fw, In, Ls, Ps, Rz, Wp	FD, SP	0.04	0.036	NA	NA	Boonma LB010
11	*Amomum repoeense* Pierre ex Gagnep.	Pud Pa	Native	W	DE	4–7	6–9	–	–	0.00	0.000	LC	LC	Boonma LB011
12	*Amomum wandokthong* (Picheans. & Yupparach) Škorničk. & Hlavatá	Wan Dok Thong	Endemic	C	HG	4–11	Not seen	Fw, In, Wp	RT	0.38	0.382	NA	NA	Boonma LB012
13	*Boesenbergia collinsii* Mood, L.M.Prince & Triboun	Kra Chai Dok Lueang	Endemic	W/C	DE, HG, LS	7–11	Not seen	Wp	RT	0.03	0.027	NE	VU B2ab (ii, v)	Boonma LB013
14	*Boesenbergia curtisii* (Baker) Schltr.	Kra Chai Tai	Native	C	HG	7–9	Not seen	Fw, Wp	RT	0.02	0.018	NA	NA	Boonma LB014
15	*Boesenbergia maxwellii* Mood, L.M.Prince & Triboun	Wan Gai Daeng	Native	C	HG	7–9	Not seen	Fw, Wp	RT	0.06	0.064	NA	NA	Boonma LB015
16	*Boesenbergia petiolata* Sirirugsa	Wan Naresuan	Native	W/C	DE, HG, LS	7–10	8–11	Fw, Wp	RT	0.08	0.082	NE	LC	Boonma LB016
17	*Boesenbergia rotunda* (L.) Mansf.	Kra Chai	Native	W/C	AG, DE, HG, LS, MX	7–9	9–10	Ls, Ps, Rs, Rz	CC, FD, SP, MC	2.61	1.000	LC	LC	Boonma LB017
18	*Boesenbergia thorelii* (Gagnep.) Loes.	Wan Phetglab	Native	C	HG	7–9	Not seen	Fw, Wp	RT	0.03	0.027	NA	NA	Boonma LB018
19	*Cornukaempferia argentifolia* Boonma & Saensouk	Proh Thong Bai Ngern	Endemic	C	HG	7–8	8–9	Wp	OM	0.15	0.155	NA	NA	Boonma LB019
20	*Cornukaempferia aurantiiflora* Mood & K.Larsen	Proh Thong	Endemic	C	HG	7–8	8–9	Wp	OM	0.17	0.173	NA	NA	Boonma LB020
21	*Cornukaempferia kamolwaniae* Picheans.	Proh Thong Kamolwan	Endemic	C	HG	7–8	8–9	Wp	OM	0.05	0.045	NA	NA	Boonma LB021
22	*Curcuma aeruginosa* Roxb.	Wan Maha Mek	Native	C	HG	3–6	Not seen	Rz, Wp	MC, RT	0.08	0.055	NA	NA	Boonma LB022
23	*Curcuma alismatifolia* Gagnep.	Pathumma, Krachiao	Native	C	HG	7–9	8–10	Wp	OM	0.65	0.655	NA	NA	Boonma LB023
24	*Curcuma amarissima* Roscoe	Khamin Dam	Native	C	HG	3–6	Not seen	Rz, Wp	MC, RT	0.08	0.055	NA	NA	Boonma LB024
25	*Curcuma angustifolia* Roxb.	Krachiao	Native	W/C	AG, HG, MX	3–9	5–10	Fw, In, Ps, Rz, Wp	CC, FD, RT	0.75	0.709	NE	LC	Boonma LB025
26	*Curcuma borealis* Saensouk, P.Saensouk & Boonma	Krachiao Thep Apsorn	Endemic	C	HG	3–5	5–7	Wp	OM	0.13	0.127	NA	NA	Boonma LB026
27	*Curcuma chantaranothaii* Boonma & Saensouk	Wan Khum Rotjana	Endemic	C	HG	7–9	8–10	Fw, In, Wp	RT	0.06	0.064	NA	NA	Boonma LB027
28	*Curcuma charanii* Boonma & Saensouk	Krachiao Charan	Endemic	W/C	DE, HG, LS, MX	7–9	8–10	Wp	RT	0.02	0.018	NE	CR B2ab (ii, v)	Boonma LB028
29	*Curcuma cinnabarina* Škorničk. & Soonthornk.	Krachiao Usa	Endemic	C	HG	7–9	8–10	Fw, In, Wp	OM, RT	0.07	0.045	NA	NA	Boonma LB029
30	*Curcuma comosa* Roxb.	Wan Chak Mot Luk	Native	C	AG, HG	3–5	5–7	Rz, Wp	MC, RT	0.17	0.155	NA	NA	Boonma LB030
31	*Curcuma globulifera* Škorničk. & Soonthornk.	Wan Salika	Endemic	C	HG	3–5	5–7	Fw, In, Wp	RT	0.15	0.145	NA	NA	Boonma LB031
32	*Curcuma harmandii* Gagnep.	Chor Morrakot	Native	W/C	DE, HG, LS, MX	7–9	8–10	Wp	OM, RT	0.12	0.073	LC	LC	Boonma LB032
33	*Curcuma involucrata* (King ex Baker) Škorničk.	Wan Dak Dae	Native	W/C	HG, LS, MX	3–5	5–7	Wp	RT	0.03	0.027	NE	LC	Boonma LB033
34	*Curcuma longa* L.	Khamin, Khamin Chan	Introduced	C	AG, HG	7–9	8–10	Fw, In, Rz	CC, CT, FD, MC, SP	2.77	1.000	NA	NA	Boonma LB034
35	*Curcuma × lopburiensis* Boonma, Saensouk & P.Saensouk (nothosp. nov.)	Siri Chandra	Endemic	W/C	DE, HG, LS, MX	8–9	Not seen	Wp	OM	0.01	0.009	NE	DD	Boonma LB035
36	*Curcuma mangga* Valeton & Zijp	Khamin Khaow	Introduced	C	AG, HG	3–5	6–8	Fw, In, Rz	CC, FD, SP, MC	1.11	0.473	NA	NA	Boonma LB036
37	*Curcuma nakhonphanomensis* Boonma, Saensouk & P.Saensouk	Maha Udon Nakhon Phanom	Native	C	HG	7–9	8–10	Fw, In, Wp	RT	0.05	0.045	NA	NA	Boonma LB037
38	*Curcuma parviflora* Wall.	Wan Thep Rum Luek	Native	W/C	DE, HG, LS, MX	7–9	8–10	Wp	OM, RT	0.69	0.491	NE	LC	Boonma LB038
39	*Curcuma peninsularis* Saensouk, P.Saensouk, Maknoi & Boonma	Ploy Andaman	Endemic	C	HG	7–9	8–10	Rz, Wp	MC, OM	0.59	0.573	NA	NA	Boonma LB039
40	*Curcuma petiolata* Roxb.	Wan Thep Prachumporn	Native	W/C	DE, HG, LS, MX	7–9	8–10	In, Wp	CC, OM	0.46	0.436	DD	LC	Boonma LB040
41	*Curcuma phrayawan* Boonma & Saensouk	Phra Ya Wan	Native	C	HG	7–9	Not seen	Rz, Wp	MC, OM, RT	0.85	0.409	NA	NA	Boonma LB041
42	*Curcuma pierreana* Gagnep.	Maha Udom Daeng	Native	C	HG	7–9	Not seen	Fw, In, Wp	RT	0.13	0.127	NA	NA	Boonma LB042
43	*Curcuma puangpeniae* Boonma & Saensouk	Wan Thep Raksa	Endemic	C	HG	7–9	Not seen	Wp	RT	0.25	0.255	NA	NA	Boonma LB043
44	*Curcuma pulcherrima* Boonma, Saensouk & P.Saensouk	Krachiao Buntharik	Endemic	C	HG	7–9	Not seen	Fw, In, Wp	RT	0.01	0.009	NA	NA	Boonma LB044
45	*Curcuma putii* Maknoi & Jenjitt.	Krachiao Lueang	Endemic	W/C	HG, LS, MX	7–9	8–10	Fw, In, Wp	FD, OM	0.24	0.191	NE	LC	Boonma LB045
46	*Curcuma rangjued* Saensouk & Boonma	Wan Rang Jued	Native	C	HG	7–9	8–10	Rz, Wp	MC, RT	0.51	0.336	NA	NA	Boonma LB046
47	*Curcuma rangsimae* Boonma & Saensouk	Bussarakham	Endemic	C	HG	7–9	8–10	Fw, In, Wp	RT	0.15	0.155	NA	NA	Boonma LB047
48	*Curcuma retrocalcaria* Saensouk, P.Saensouk & Boonma	Krachiao Sri Sunthorn	Endemic	C	HG	7–9	Not seen	Fw, In, Wp	RT	0.02	0.018	NA	NA	Boonma LB048
49	*Curcuma rhabdota* Sirirugsa & M.F.Newman	Bua Lai Ubon	Native	C	HG	7–9	8–10	Wp	OM	0.25	0.245	NA	NA	Boonma LB049
50	*Curcuma roscoeana* Wall.	Krachiao Som	Native	C	HG	7–9	8–10	Wp	OM	0.15	0.145	NA	NA	Boonma LB050
51	*Curcuma rosea* P.Saensouk, Saensouk & Boonma	Wan Umawadi	Endemic	C	HG	7–9	8–10	Fw, In, Wp	OM, RT	0.22	0.136	NA	NA	Boonma LB051
52	*Curcuma roseobracteata* P.Saensouk, Saensouk, Maknoi & Boonma	Wan Ngu Hao Chomphoo	Endemic	C	HG	7–9	8–10	Fw, In, Wp	OM, RT	0.20	0.100	NA	NA	Boonma LB052
53	*Curcuma rubescens* Roxb.	Wan Maha Prab	Native	C	HG	4–6	Not seen	Rz, Wp	MC, OM, RT	0.58	0.373	NA	NA	Boonma LB053
54	*Curcuma rubrobracteata* Škorničk., M.Sabu & Prasanthk.	Wan Ngu Hao Daeng	Native	C	HG	7–9	Not seen	Fw, In, Wp	RT	0.20	0.200	NA	NA	Boonma LB054
55	*Curcuma saraburiensis* Boonma & Saensouk	Saraburi Ram Luek	Endemic	W/C	DE, HG, LS, MX	7–9	8–10	Fw, In, Wp	CC, OM, RT	0.03	0.009	NE	EN B2ab (ii, v)	Boonma LB055
56	*Curcuma siamensis* Saensouk & Boonma	Khamin Siam	Endemic	C	HG	7–9	8–10	Fw, In, Wp	RT	0.04	0.036	NA	NA	Boonma LB056
57	*Curcuma singularis* Gagnep.	Krachiao Khaow, Dok Din	Native	W/C	HG, LS, MX	3–5	6–8	Fw, In, Rz, Wp	CC, FD, RT	0.93	0.645	NE	LC	Boonma LB057
58	*Curcuma suphanensis* P.Saensouk, Boonma, Rakarcha, Maknoi, Wongnak & Saensouk	Krachiao Suphan	Endemic	C	HG	7–9	8–10	Fw, In, Wp	RT	0.03	0.027	NA	NA	Boonma LB058
59	*Curcuma supraneeana* (W.J.Kress & K.Larsen) Škorničk.	Krachiao Supranee	Endemic	W/C	HG, LS, MX	7–9	8–10	Wp	OM, RT	0.48	0.464	CR B1ab (iii)	CR B1ab (iii, v)	Boonma LB059
60	*Curcuma suraponii* Boonma	Wan Krabi Thong	Endemic	C	HG	7–9	8–10	Fw, In, Wp	OM, RT	0.27	0.164	NA	NA	Boonma LB060
61	*Curcuma thorelii* Gagnep.	Krachiao Khaow	Native	W/C	DE, HG, LS, MX	7–9	8–10	Wp	OM, RT	0.60	0.509	NE	LC	Boonma LB061
62	*Curcuma ubonensis* Boonma, Saensouk, Maknoi & P.Saensouk	Krachiao Ubon	Endemic	C	HG	7–9	8–10	Wp	RT	0.03	0.027	NA	NA	Boonma LB062
63	*Curcuma wanchaii* Saensouk, P.Saensouk, Maknoi & Boonma	Krachiao Wanchai	Endemic	C	HG	7–9	8–10	Wp	OM	0.04	0.036	NA	NA	Boonma LB063
64	*Curcuma wanenlueanga* Saensouk, Thomudtha & Boonma	Wan En Lueang	Native	C	HG	7–9	8–10	Rz, Wp	MC, RT	0.22	0.200	NA	NA	Boonma LB064
65	*Curcuma zanthorrhiza* Roxb.	Wan Chak Mot Luk	Introduced	C	HG	7–9	8–10	Rz, Wp	MC, RT	0.08	0.073	NA	NA	Boonma LB065
66	*Etlingera elatior* (Jack) R.M.Sm.	Da Lah	Native	C	AG, HG	1–12	1–12	In, Br, Wp	CC, FD, OM	1.04	0.891	NA	NA	Boonma LB066
67	*Gagnepainia godefroyi* (Baill.) K.Schum.	Wan Phet Na Thang	Native	W/C	DE, HG, LS, MX	3–5	Not seen	Fw, In, Wp	RT	0.03	0.027	LC	LC	Boonma LB067
68	*Globba chrysochila* Sangvir. & M.F.Newman	Khao Phansa	Endemic	W/C	DE, HG, LS, MX	7–9	8–10	In, Wp	CC, OM	0.06	0.045	NE	LC	Boonma LB068
69	*Globba colpicola* K.Schum.	Khao Phansa Puang Thong	Endemic	W/C	DE, HG, LS, MX	7–9	8–10	In, Wp	CC, OM	0.31	0.209	EN B1ab (iii)	EN B1ab (iii, v)	Boonma LB069
70	*Globba hilaris* Sangvir.	Khao Phansa Puang Khaow	Native	W/C	DE, HG, LS, MX	7–9	8–10	In, Wp	CC, OM	0.11	0.064	NE	LC	Boonma LB070
71	*Globba marantina* L.	Khao Phansa Phum Khao Bin	Native	W/C	DE, HG, LS, MX	7–9	8–10	In, Wp	CC, OM	0.06	0.036	LC	LC	Boonma LB071
72	*Globba rosea* Gagnep.	Khao Phan Sa	Native	C	HG	7–9	8–10	In, Wp	CC, OM	0.10	0.055	NA	NA	Boonma LB072
73	*Globba schomburgkii* Hook.f.	Khao Phan Sa	Native	W/C	DE, HG, LS, MX	7–9	8–10	In, Wp	CC, OM, RT	0.27	0.218	NE	LC	Boonma LB073
74	*Globba sherwoodiana* W.J.Kress & V.Gowda	Khao Phan Sa Khaow Phamar	Native	C	HG	7–9	8–10	In, Wp	CC, OM, RT	0.11	0.073	NA	NA	Boonma LB074
75	*Globba substrigosa* King ex Baker	Khao Phansa Rong Bai Ngern	Native	C	HG	7–9	8–10	Wp	OM	0.02	0.018	NA	NA	Boonma LB075
76	*Globba williamsiana* M.F.Newman & Sangvir.	Khao Phansa	Endemic	C	AG, HG	7–9	8–10	In, Wp	CC, OM, RT	0.50	0.382	NA	NA	Boonma LB076
77	*Globba xantholeuca* Craib	Khao Phansa	Endemic	W/C	HG, LS, MX	7–9	8–10	In, Wp	CC, OM	0.05	0.027	LC	LC	Boonma LB077
78	*Hedychium coronarium* J.Koenig	Maha Hong Khaow	Native	C	AG, HG	7–9	8–10	Wp	OM, RT	0.45	0.400	NA	NA	Boonma LB078
79	*Hedychium flavescens* Carey ex Roscoe	Maha Hong Lueang	Introduced	C	HG	7–9	8–10	Wp	OM	0.06	0.064	NA	NA	Boonma LB079
80	*Kaempferia angustifolia* Roxb.	Wan Prab Samut	Native	C	HG	7–9	8–10	Rz, Wp	MC, RT	0.10	0.073	NA	NA	Boonma LB080
81	*Kaempferia chaveerachiae* Saensouk, P.Saensouk & Boonma	Proh Chom Chandra	Endemic	W/C	DE, HG, LS, MX	7–9	8–10	Wp	RT	0.01	0.009	NE	CR B2ab (iii),D1	Boonma LB081
82	*Kaempferia elegans* Wall.	Wan Nok Khum	Native	C	AG, HG	7–9	8–10	Wp	CC, OM, RT	0.84	0.555	NA	NA	Boonma LB082
83	*Kaempferia galanga* L.	Proh Hom	Native	C	HG	7–9	8–10	Ls, Ps, Rz, Wp	CC, FD, MC	0.20	0.127	NA	NA	Boonma LB083
84	*Kaempferia gilbertii* W.Bull	Wan Maha Ni Yom	Introduced	C	HG	7–9	8–10	Wp	CC, OM, RT	0.73	0.500	NA	NA	Boonma LB084
85	*Kaempferia larsenii* Sirirugsa	Wan Gai Kuk	Endemic	C	HG	7–9	8–10	Wp	RT	0.07	0.073	NA	NA	Boonma LB085
86	*Kaempferia lopburiensis* Picheans.	Proh Lopburi	Endemic	W/C	DE, HG, LS, MX	3–5	5–7	Wp	OM	0.04	0.036	NE	EN B2ab (iii, v), D1	Boonma LB086
87	*Kaempferia maculifolia* Boonma & Saensouk	Proh Bai Lai Jut	Endemic	W/C	DE, HG, LS, MX	7–9	8–10	Wp	RT	0.05	0.045	NE	EN B2ab (ii, iv)	Boonma LB087
88	*Kaempferia marginata* Carey ex Roscoe	Wan Toob Moob	Native	W/C	HG, LS, MX	7–9	8–10	Ls, Rz, Wp	CC, FD, MC, RT	0.43	0.309	NE	LC	Boonma LB088
89	*Kaempferia napavarniae* Saensouk, P.Saensouk & Boonma	Proh Napavarn	Endemic	W/C	HG, LS, MX	7–9	8–10	Wp	RT	0.02	0.018	NE	EN B2ab (ii, v)	Boonma LB089
90	*Kaempferia pardi* K.Larsen & Jenjitt.	Proh Suea Dao	Endemic	W/C	DE, HG, LS, MX	7–9	8–10	Wp	OM	0.06	0.064	NE	EN B2ab (ii, v)	Boonma LB090
91	*Kaempferia parviflora* Wall. ex Baker	Krachai Dam	Native	C	AG, HG	7–9	8–10	Rz, Wp	CC, MC, RT	0.46	0.218	NA	NA	Boonma LB091
92	*Kaempferia pulchra* Ridl.	Proh Pa, Proh Daang	Native	C	AG, HG	7–9	8–10	Wp	CC, OM	0.19	0.164	NA	NA	Boonma LB092
93	*Kaempferia roscoeana* Wall.	Proh Pa Dok Khaow	Native	W/C	DE, HG, LS, MX	7–9	8–10	Wp	OM	0.06	0.045	NE	LC	Boonma LB093
94	*Kaempferia rotunda* L.	Wan Thip Pha Ya Nate	Native	W/C	DE, HG, LS, MX	3–5	5–7	Fw, Ps, Rz, Wp	CC, MC, OM, RT	0.76	0.345	NE	LC	Boonma LB094
95	*Kaempferia sakolchaii* P.Saensouk, Saensouk & Boonma	Proh Sakolchai	Endemic	C	HG	7–9	8–10	Wp	RT	0.02	0.018	NA	NA	Boonma LB095
96	*Kaempferia sakonensis* Saensouk, P.Saensouk & Boonma	Proh Sakon Nakhon	Endemic	C	HG	7–9	8–10	Wp	RT	0.02	0.018	NA	NA	Boonma LB096
97	*Kaempferia saraburiensis* Picheans.	Proh Saraburi	Endemic	W/C	DE, HG, LS, MX	7–9	8–10	Wp	OM	0.06	0.064	NE	CR C2a (i, ii), D1	Boonma LB097
98	*Kaempferia unifolia* Saensouk & P.Saensouk	Proh Bai Diaw	Endemic	C	HG	7–9	8–10	Wp	RT	0.02	0.018	NA	NA	Boonma LB098
99	*Meistera koenigii* (J.F.Gmel.) Škorničk. & M.F.Newman	Reo Puang A-Ngun	Native	W	DE, LS, MX	4–6	5–7	–	–	0.00	0.000	NE	LC	Boonma LB099
100	*Meistera tomrey* (Gagnep.) Škorničk. & M.F.Newman	Reo Pa	Native	W	DE, LS, MX	3–6	5–7	–	–	0.00	0.000	LC	LC	Boonma LB100
101	*Wurfbainia uliginosa* (J.Koenig) Giseke	Krawan Pa	Native	W	MX	4–7	5–8	–	–	0.00	0.000	LC	LC	Boonma LB101
102	*Wurfbainia vera* (Blackw.) Škorničk. & A.D.Poulsen	Krawan	Native	C	HG	4–5	6–8	Fs	FD, SP	0.07	0.073	NA	NA	Boonma LB102
103	*Zingiber brachystachys* Triboun & K.Larsen	Khing Phra Phutthabat	Endemic	W/C	DE, HG, LS, MX	7–9	8–10	Wp	RT	0.01	0.009	NE	EN B2ab (iv, v), D1	Boonma LB103
104	*Zingiber citriodorum* Theilade & Mood	Ta Krai Pran	Endemic	C	HG	4–5	6–8	Rz, Wp	MC, RT	0.06	0.036	NA	NA	Boonma LB104
105	*Zingiber officinale* Roscoe	Khing	Introduced	C	AG, HG	7–9	8–10	Fw, In, Ls, Ps, Rz	CC, CT, FD, SP, MC	2.63	1.000	NA	NA	Boonma LB105
106	*Zingiber ottensii* Valeton	Plai Dam	Introduced	C	HG	7–9	8–10	Fw, Rz, Wp	MC, RT	0.24	0.164	NA	NA	Boonma LB106
107	*Zingiber parishii* Hook.f.	Wan Hom	Native	W/C	DE, HG, LS, MX	5–7	6–9	Wp	OM	0.03	0.027	NE	LC	Boonma LB107
108	*Zingiber purpureum* Roscoe	Plai, Plai Lueang	Introduced	C	AG, HG	7–9	8–10	Fw, In, Ls, Ps, Rz, Wp	CC, CT, MC, RT	0.54	0.255	NA	NA	Boonma LB108
109	*Zingiber thorelii* Gagnep.	Khing Dok Din	Native	W/C	DE, HG, LS, MX	7–9	8–10	Fw, In	FD	0.06	0.064	LC	LC	Boonma LB109
110	*Zingiber zerumbet* (L.) Roscoe ex Sm.	Kra Tue	Native	W/C	DE, HG, LS, MX	7–9	8–10	Fw, In, Ls, Ps, Rz, Wp	FD, MC, RT	0.17	0.109	DD	LC	Boonma LB110

^1^ Occurrence type: wild populations recorded in natural habitats (W); cultivated (C); recorded both as wild populations and cultivated (W/C). ^2^ Habitats: agricultural area (AG); dry evergreen forest (DE); home garden (HG); limestone area (LS); mixed deciduous forest (MX). ^3^ Months: January (1); February (2); March (3); April (4); May (5); June (6); July (7); August (8); September (9); October (10); November (11); December (12). ^4^ Used parts: fruit and seeds (Fs); flowers (Fw); inflorescences (In); leaves (Ls); pseudostem (Ps); roots (Rs); rhizome (Rz); whole plant (Wp). ^5^ Purposes: commercial cultivation (CC); cosmetics (CT); food (FD); medicine (MC); ornamental (OM); ritual uses and beliefs (RT); spice (SP). ^6^ Conservation status: critically endangered (CR); endangered (EN); vulnerable (VU); least concern (LC); data deficient (DD); not evaluated (NE) [36,37]. NA = Not assessed, as the taxon is cultivated and not considered part of the wild flora of Lop Buri Province. The total number of taxa includes wild, cultivated, introduced, and locally utilized taxa, and should not be interpreted as the number of wild taxa only.

**Table 2:** Informant Consensus Factor (Fic) values for therapeutic categories associated with medicinal uses of Zingiberaceae in Lop Buri Province, Thailand.

Therapeutic Groups	Nur	Nt	Fic
Infections	4	1	1.000
Musculoskeletal and joint disorders	55	7	0.889
Gastrointestinal disorders	120	19	0.849
Antipyretic	63	11	0.839
Eye disorders	13	3	0.833
Poisoning and toxicological conditions	29	6	0.821
Obstetric, gynecological and urinary disorders	48	10	0.809
Skin disorders	57	12	0.804
Cardiovascular disorders	18	6	0.706
Respiratory disorders	13	5	0.667
Nutrition and blood-related disorders	27	10	0.654

**Table 3:** Comparative morphological characters of *Curcuma saraburiensis*, *C. × lopburiensis*, and *C. parviflora.*

Character	*C. saraburiensis*	*C. × lopburiensis*	*C. parviflora*
Rhizome	Ovoid, Internally light brown	Short ovoid, Internally cream to pale buff	Ovoid to subglobose, Internally cream to pale orange or pink
Tuberous roots	Globose to subglobose	Ovoid to ellipsoid	Ovoid, ellipsoid to fusiform
Leafy shoot height	30–50 cm	30–50 cm	15–50 cm
Number of leaves	4–7	3–5	2–6
Leaf blade shape	Elliptic to ovate	Elliptic to narrowly ovate	Ovate to elliptic
Leaf blade size	16–22 × 7–9 cm	13–26 × 5.5–12 cm	7–25 × 3–8 cm
Leaf base	Obtuse to attenuate	Cuneate–oblique to rounded	Cuneate to rounded
Lower leaf surface indumentum	Pubescent	Glabrous or puberulent	Glabrous
Leaf margin	Slightly undulate	Slightly undulate	Slightly undulate
Petiole length	8–14 cm	4–12 cm	1.5–30 cm
Inflorescence position	Terminal	Terminal	Terminal
Peduncle length	20–25 cm	10–20 cm	5–15(–20) cm
Thyrse length	8–12 cm	7–9 cm	4–10 cm
Coma bracts	White with green edges	Pale green or white with green edges, or white with green apex	White, often with green apex
Fertile bracts	Light green with pale green or white longitudinal lines and two white circular patches	Plain green or with patterns similar to those of *C. saraburiensis*	Plain green
Fertile bract apex	Rounded	Obtuse to rounded	Acute to rounded
Flowers per bract	3–7	4–6	2–4
Flower type	Open type	Intermediate between open and gullet type	Gullet type
Flower length	3.5–4.0 cm	3.0–3.2 cm	1.7–2.5(–3.0) cm
Calyx length	15–18 mm	8–10 mm	4–7 mm
Calyx apex	Trilobed	Trilobed	Trilobed
Lateral staminodes	White with 3–4 dark red lines at base	White to pale purple, with 2–3 pale reddish lines at base	White, sometimes with pale violet tinge
Labellum coloration	White with purple patches and dark red lines at base	White centrally with purple distal part and pale reddish lines at base, sometimes with pale yellow patch at the basal–central part	White basally, purple to violet distally
Labellum apex	Deeply bilobed	Deeply bilobed with slightly crisped margins	Bilobed, crisped to fringed margins
Labellum incision	Up to 9 mm	Up to 5 mm	Shallowly bilobed
Anther	Spurless	Spurless	Spurless
Anther crest	Rounded	Rounded	Rounded
Fruit observed	Present	Not observed	Not observed

## Data Availability

The original contributions presented in this study are included in the article; further inquiries can be directed to the corresponding author.

## Appendix A

**Table A1:** Quantitative vegetative and floral characters of *Curcuma saraburiensis*, *C*. × *lopburiensis*, and *C. parviflora* used in the morphometric Principal Component Analysis (PCA).

Species	Leafy Shoot Height (cm)	Rhizome Height (cm)	Rhizome Diameter (cm)	Number of Leaves per Shoot	Minimum Leaf Blade Length (cm)	Maximum Leaf Blade Length (cm)	Minimum Leaf Blade Width (cm)	Maximum Leaf Blade Width (cm)	Minimum Petiole Length (cm)	Maximum Petiole Length (cm)	Peduncle Length (cm)	Thyrse Length (cm)	Fertile Bract Length (cm)	Fertile Bract Width (cm)	Calyx Length (cm)	Floral Tube Length (cm)	Labellum Length (cm)	Labellum Width (cm)	Labellum Incision Depth (mm)	Lateral Staminode Length (cm)	Lateral Staminode Width (cm)	Anther Length (mm)
*C. saraburiensis* 1	30	2	1	4	15	22	5	7	8	13	20	8	2.0	2.0	1.5	1.7	1.2	0.9	7	1.3	0.2	5
*C. saraburiensis* 2	32	2	1	4	16	21	6	8	8	13.5	23	12	2.5	2.3	1.6	1.9	1.3	1.0	8	1.4	0.3	6
*C. saraburiensis* 3	30	2	1	4	15	23	5	7	8	13	22	9	2.0	2.0	1.5	1.8	1.2	0.9	8	1.4	0.3	6
*C. saraburiensis* 4	35	2.5	1.5	5	17	22	5.3	8	8.5	14	20	11	2.2	2.2	1.6	1.9	1.3	1.0	7	1.3	0.2	5
*C. saraburiensis* 5	37	3	2	6	16	22	5	8	8	14	22	12	2.5	2.5	1.7	2	1.3	1.0	8	1.4	0.3	6
*C. saraburiensis* 6	35	2.5	1.5	5	15	23	5.2	7	8	13	22	9	2.0	2.0	1.5	1.8	1.2	0.9	8	1.4	0.3	5.5
*C. saraburiensis* 7	32	2.5	1.5	5	15	22	6	8	8	12.5	20	12	2.5	2.4	1.7	2	1.3	1.0	8	1.4	0.3	6
*C. saraburiensis* 8	38	3	2	6	18	22	6.4	8	8.5	14	20	10	2.2	2.2	1.6	1.9	1.3	1.0	7	1.3	0.2	5
*C. saraburiensis* 9	39	3	2	5	17	24	7	8	8	14.5	22	11	2.3	2.3	1.6	1.9	1.3	1.0	8	1.4	0.3	5.5
*C. saraburiensis* 10	45	3	2	6	18	24	7	9	9	15	24	12	2.5	2.4	1.7	2	1.4	1.0	8	1.4	0.3	6
*C. saraburiensis* 11	50	3	2	6	16	27	8	9	11	15	25	12	2.5	2.5	1.8	2	1.4	1.0	9	1.5	0.3	6
*C. saraburiensis* 12	43	3	2	6	14	23	7	9	9	14	23	11	2.3	2.3	1.6	1.9	1.4	1.0	8	1.4	0.3	6
*C. saraburiensis* 13	41	3	2	5	15	22	7	9	9	14	22	10	2.2	2.2	1.6	1.8	1.4	1.0	8	1.4	0.3	6
*C. saraburiensis* 14	40	3	2	7	15	23	7	9	8	13.5	24	12	2.5	2.5	1.8	2	1.4	1.0	7	1.3	0.2	6
*C. saraburiensis* 15	38	3	2	5	16	22	5	8	8.5	14	22	11	2.3	2.3	1.6	1.9	1.3	1.0	7	1.3	0.2	5
*C. saraburiensis* 16	39	3	2	5	16	22	5	8	8	13	21	10	2.2	2.1	1.6	1.9	1.3	1.0	8	1.4	0.2	5
*C. saraburiensis* 17	46	3	2	6	17	24	7	9	9	14	22	11	2.2	2.2	1.5	1.7	1.2	0.9	7	1.4	0.3	5.5
*C. saraburiensis* 18	40	3	2	6	15	24	7	9	8	14	22	10	2.0	2.0	1.5	1.8	1.4	1.0	8	1.4	0.3	6
*C. saraburiensis* 19	38	3	2	6	16	24	6	8	8.5	14.5	25	12	2.5	2.3	1.6	1.9	1.3	1.0	7	1.4	0.3	5.5
*C. saraburiensis* 20	37	3	2	5	17	23	6	8	8	13	22	11	2.3	2.2	1.5	1.7	1.2	0.9	7	1.3	0.2	5
*C. × lopburiensis* 1	38	2.4	1.2	4	13	22	5.5	8	5	7.3	15	7.8	2.4	1.7	0.9	2.0	1.2	0.9	5	1.2	0.3	4
*C. × lopburiensis* 2	46	2.9	1.5	5	17	24.5	8	11	8	11.5	19	8.9	2.9	2.4	1.0	2.0	1.3	1.0	5	1.3	0.4	5
*C. × lopburiensis* 3	30	2.0	1.0	3	16.3	23	6	9.5	4	5.8	12	7.1	2.1	1.3	0.8	2.0	1.1	0.8	5	1.0	0.3	4
*C. × lopburiensis* 4	42	2.6	1.3	4	15	24	7	10	7	8.9	17	8.3	2.6	2.0	0.9	2.0	1.3	0.9	5	1.3	0.35	4
*C. × lopburiensis* 5	50	3.0	1.5	5	18	26.0	8	12.0	10	12.0	20	9.0	3.0	2.5	1.0	2.0	1.3	1.0	5	1.3	0.4	5
*C. parviflora* 1	19	1.1	1.0	2	10	15.8	2	3.4	5	13.5	6	4.8	1.6	1.3	0.4	1.1	0.8	0.5	2	0.6	0.15	3
*C. parviflora* 2	31	1.4	1.3	4	14.7	17.3	3	4.9	8.5	18.5	9	6.7	2.1	1.9	0.5	1.3	0.85	0.65	2	0.72	0.22	3
*C. parviflora* 3	45	1.8	1.7	5	22.4	25	4	6.9	12	23.0	13	9.1	2.7	2.7	0.7	1.6	1.0	0.75	3	0.8	0.3	3
*C. parviflora* 4	24	1.2	1.2	3	11.5	16	2	4.1	7	15.4	7	5.4	1.9	1.6	0.5	1.2	0.8	0.6	2	0.65	0.18	3
*C. parviflora* 5	38	1.6	1.5	5	14	18.2	3	5.8	10	14.2	11	8.0	2.4	2.3	0.6	1.5	0.9	0.7	3	0.77	0.25	3
*C. parviflora* 6	28	1.3	1.3	4	13.1	18	2	4.4	12	17.2	8	6.0	2.0	1.8	5	1.3	0.85	0.6	2	0.68	0.2	3
*C. parviflora* 7	49	1.9	1.9	6	19	24.8	4	7.6	19	28.4	18	9.8	2.9	2.9	7	1.7	1.0	0.8	3	0.8	0.35	3
*C. parviflora* 8	35	1.5	1.4	4	12	16.5	3	5.3	5	10.7	10	7.2	2.3	2.1	5	1.4	0.9	0.65	2	0.75	0.24	3
*C. parviflora* 9	21	1.1	1.1	3	15.4	18	2	3.7	6	13.6	6	5.0	1.7	1.4	4	1.2	0.8	0.55	2	0.6	0.15	3
*C. parviflora* 10	42	1.7	1.7	5	16	20.7	4	6.4	12	19.3	12	8.7	2.6	2.5	6	1.5	0.95	0.72	3	0.79	0.28	3
*C. parviflora* 11	26	1.3	1.2	3	12.3	15	3	4.3	7	16.2	8	5.8	2.0	1.7	5	1.3	0.85	0.6	2	0.67	0.2	3
*C. parviflora* 12	47	1.9	1.8	6	16	23.5	4	7.1	12	25.6	15	9.4	2.8	2.8	7	1.6	1.0	0.78	3	0.8	0.32	3
*C. parviflora* 13	33	1.4	1.4	4	15.8	19	2	5.1	9.8	19.8	10	7.0	2.2	2.0	5	1.4	0.9	0.65	2	0.74	0.23	3
*C. parviflora* 14	17	1.0	1.0	2	13.8	16	2	3.2	5	11.8	5	4.3	1.5	1.2	4	1.1	0.8	0.5	2	0.55	0.15	3
*C. parviflora* 15	39	1.6	1.6	5	14	18.9	4	6.0	7.8	15.1	11	8.2	2.4	2.3	6	1.5	0.9	0.7	3	0.77	0.25	3
*C. parviflora* 16	29	1.3	1.3	4	13.9	17	3	4.7	8.5	17.8	8	6.3	2.1	1.9	5	1.3	0.85	0.62	2	0.7	0.21	3
*C. parviflora* 17	50	2.0	2.0	6	16	25.0	5	8.0	22	28	20	10.0	3.0	3.0	7	1.7	1.0	0.8	3	0.8	0.35	3
*C. parviflora* 18	36	1.5	1.5	4	12.5	17.1	3	5.4	8	11.6	10	7.4	2.3	2.1	5	1.4	0.9	0.68	2	0.75	0.24	3
*C. parviflora* 19	23	1.2	1.1	3	10.2	18.2	2	3.9	4.5	14.5	7	5.2	1.8	1.5	5	1.2	0.8	0.58	2	0.63	0.17	3
*C. parviflora* 20	43	1.7	1.7	5	17	21.5	4	6.6	15	20.8	12	8.8	2.6	2.5	6	1.5	0.95	0.73	3	0.79	0.29	3
